# Nodal signaling is required for mesodermal and ventral but not for dorsal fates in the indirect developing hemichordate, *Ptychodera flava*

**DOI:** 10.1242/bio.011809

**Published:** 2015-05-15

**Authors:** Eric Röttinger, Timothy Q. DuBuc, Aldine R. Amiel, Mark Q. Martindale

**Affiliations:** 1 Université Nice Sophia Antipolis, IRCAN, UMR 7284, 06107 Nice, France; 2 CNRS, IRCAN, UMR 7284, 06107 Nice, France; 3 INSERM, IRCAN, U1081, 06107 Nice, France; 4 The Whitney Marine Laboratory for Marine Science, University of Florida, St. Augustine, FL 32080-8610, USA

**Keywords:** Ambulacraria, Dorsoventral axis, Evolution, Hemichordate, Mesoderm, Nodal pathway

## Abstract

Nodal signaling plays crucial roles in vertebrate developmental processes such as endoderm and mesoderm formation, and axial patterning events along the anteroposterior, dorsoventral and left-right axes. In echinoderms, Nodal plays an essential role in the establishment of the dorsoventral axis and left-right asymmetry, but not in endoderm or mesoderm induction. In protostomes, Nodal signaling appears to be involved only in establishing left-right asymmetry. Hence, it is hypothesized that Nodal signaling has been co-opted to pattern the dorsoventral axis of deuterostomes and for endoderm, mesoderm formation as well as anteroposterior patterning in chordates. Hemichordata, together with echinoderms, represent the sister taxon to chordates. In this study, we analyze the role of Nodal signaling in the indirect developing hemichordate *Ptychodera flava.* In particular, we show that during gastrulation *nodal* transcripts are detected in a ring of cells at the vegetal pole that gives rise to endomesoderm and in the ventral ectoderm at later stages of development. Inhibition of Nodal function disrupts dorsoventral fates and also blocks formation of the larval mesoderm. Interestingly, molecular analysis reveals that only mesodermal, apical and ventral gene expression is affected while the dorsal side appears to be patterned correctly. Taken together, this study suggests that the co-option of Nodal signaling in mesoderm formation and potentially in anteroposterior patterning has occurred prior to the emergence of chordates and that Nodal signaling on the ventral side is uncoupled from BMP signaling on the dorsal side, representing a major difference from the molecular mechanisms of dorsoventral patterning events in echinoderms.

## INTRODUCTION

Hemichordata is a group of marine worms that together with its sister taxon Echinodermata, form a clade called Ambulacraria within the deuterostomes that form the sister group to chordates (Metschnikoff, 1881; Swalla and Smith, 2008). Fate-mapping experiments have shown that the cleavage patterns, as well as the early fate maps of direct and indirect developing hemichordates, are similar to those of indirect-developing echinoids (Colwin and Colwin, 1951; Cameron et al., 1987; Cameron et al., 1989; Cameron and Davidson, 1991; Henry et al., 2001). While the bilaterally symmetric echinoderm larvae exhibit strong similarities to chordates in axial patterning and germ layer specification events, adult body plan comparisons in echinoderms have been difficult due to their unique adult pentaradial symmetry. However, both the larval and adult body plans of enteropneust hemichordates are bilaterally symmetric, and larvae from indirect developing hemichordates such as *Ptychodera flava* (*P. flava*) share similarities in morphology, axial organization, and developmental fate map with indirect developing echinoderms. Therefore, indirect developing hemichordates are valuable organisms for comparisons with echinoderms and chordates providing insight into the early origins of deuterostome and chordate evolution (reviewed in (Röttinger and Lowe, 2012).

The diffusible ligand Nodal has been studied in both protostomes and deuterostomes and activates the TGF-β signaling pathway and plays an important role during embryonic development including endomesoderm and patterning of the D/V, L/R and A/P axes (Duboc and Lepage, 2008; Schier, 2009; Shen, 2007). During early vertebrate development, Nodal signaling is necessary and sufficient for the induction of mesoderm and endoderm, as both tissue types are absent in animals in which Nodal function is impaired (Conlon et al., 1994; Feldman et al., 1998; Kimelman, 2006; Osada and Wright, 1999; Stainier, 2002). Nodal genes in zebrafish (*squint*) and amphioxus are expressed along the dorsal surface of the embryo during early cleavage stages and knock-down experiments ventralize embryos (Gore et al., 2005) (Onai et al., 2010; Yu et al., 2002). These findings show a role of Nodal signaling in patterning the dorsoventral (D/V) axis. In vertebrates, *nodals* are also expressed in structures on the left side of the embryos (such as the left lateral plate mesoderm) and the activity of this pathway is required for patterning the left-right (L/R) axis (Levin et al., 1995; Lohr et al., 1997). Deactivation of Nodal signaling causes, among other phenotypes, the inversion or the randomization of internal organs (Bamford et al., 2000; Concha et al., 2000; Yan et al., 1999). Nodal signaling has also been shown to play a crucial role in patterning the anterior-posterior (A/P) axis. In mice and zebrafish, the absence of Nodal blocks the formation of both the anterior visceral endoderm and the anterior central nervous system (Norris and Robertson, 1999; Rebagliati et al., 1998; Sampath et al., 1998; Varlet et al., 1997). Thus, Nodal plays multiple important roles in global patterning and cell type specification in vertebrates. We are interested in understanding the evolution of the various roles that Nodal signaling plays in animal development by looking at earlier diverging members of the deuterostome clade.

Although vertebrates have multiple copies of Nodal genes (Loose and Patient, 2004), echinoderms appear to have only one *nodal* gene that is expressed exclusively within ventral territories throughout embryonic development (Duboc et al., 2004; Smith et al., 2008). When Nodal signaling is impaired in both direct and indirect developing echinoderms, endoderm and mesoderm form, however, the patterning of these two germ layers is severely affected (Duboc et al., 2010). In addition, the establishment of both the D/V and the L/R axes are perturbed (Bessodes et al., 2012; Duboc et al., 2004; Duboc et al., 2005; Flowers et al., 2004; Saudemont et al., 2010; Smith et al., 2008; Su et al., 2009). Interestingly, Nodal signaling on the ventral side is not only required to specify ventral fates, but also for expression of *bmp2/4* in the ventral ectoderm (Duboc et al., 2004). Bmp2/4 diffuses to the dorsal side of the embryo where it acts to specify dorsal fates (Lapraz et al., 2009a). While a detailed GRN for D/V patterning of the echinoderm larval ectoderm has recently been proposed (Su et al., 2009); (Saudemont et al., 2010), the role of this pathway in hemichordates has yet to be explored which is required to determine the role of Nodal signaling at the base of the Deuterostomia.

In a previous study, we analyzed the molecular mechanism underlying patterning of the D/V axis of the indirect developing hemichordate *P. flava.* We showed that NiCl_2_ “ventralizes” treated hemichordate embryos and induces the formation of a circumferential mouth (Röttinger and Martindale, 2011). This is similar to what has been observed in echinoderms (Agca et al., 2009; Di Bernardo et al., 1999; Duboc et al., 2004; Hardin et al., 1992; Lallier, 1956; Minsuk and Raff, 2005), and NiCl_2_ has been shown to induce the radialized expression of *nodal* (Duboc et al., 2004). The ventrally expressed genes *chordin*, *foxA* and *bra* are known downstream targets of Nodal signaling in echinoderms (Saudemont et al., 2010) and transcripts of all three genes are also detected in ventral domains in *P. flava* (Röttinger and Martindale, 2011; Tagawa et al., 1998a; Taguchi et al., 2000)*,* suggesting that a NiCl_2_-sensitive and potentially Nodal dependent mechanism may be required to define ventral domains and pattern the dorsoventral axis in hemichordates. In the present study we analyzed the expression and role of Nodal signaling and its potential interactions with Erk and Bmp2/4 signaling during embryonic and larval development of the indirect developing hemichordate *P. flava*.

## RESULTS

### Identification of novel tissue and region specific markers in *Ptychodera flava*

**Fig. 1.**
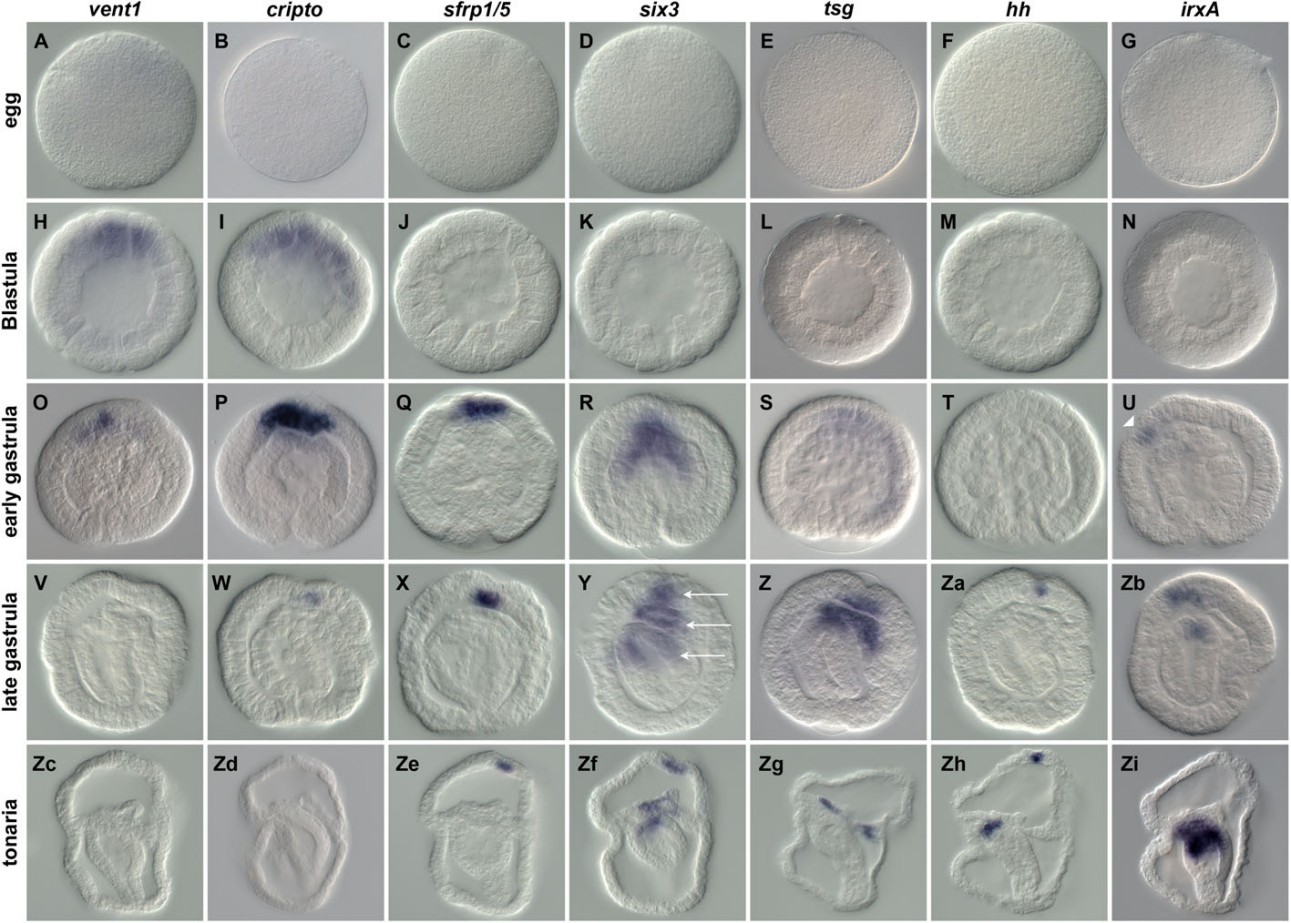
**Spatial expression of novel tissue specific markers in *Ptychodera flava*.** Spatial distribution of *Pf-vent1*, *Pf-cripto, Pf-sfrp1/5*, *Pf-six3*, *Pf-hh*, *Pf-tsg* and *Pf-irxA* transcripts during normal development analyzed by WISH. Expression of the inter apical-stomodeum domain expression of *Pf-irxA* is indicated by a single white arrowhead in (U). Ectodermal, mesodermal and endodermal expression domains of *Pf-six3* are indicated by white arrows in (Y). All embryos in this and following figures are oriented animal to the top, vegetal to the bottom, ventral to the left and dorsal to the right if not stated otherwise.

**Fig. 2.**
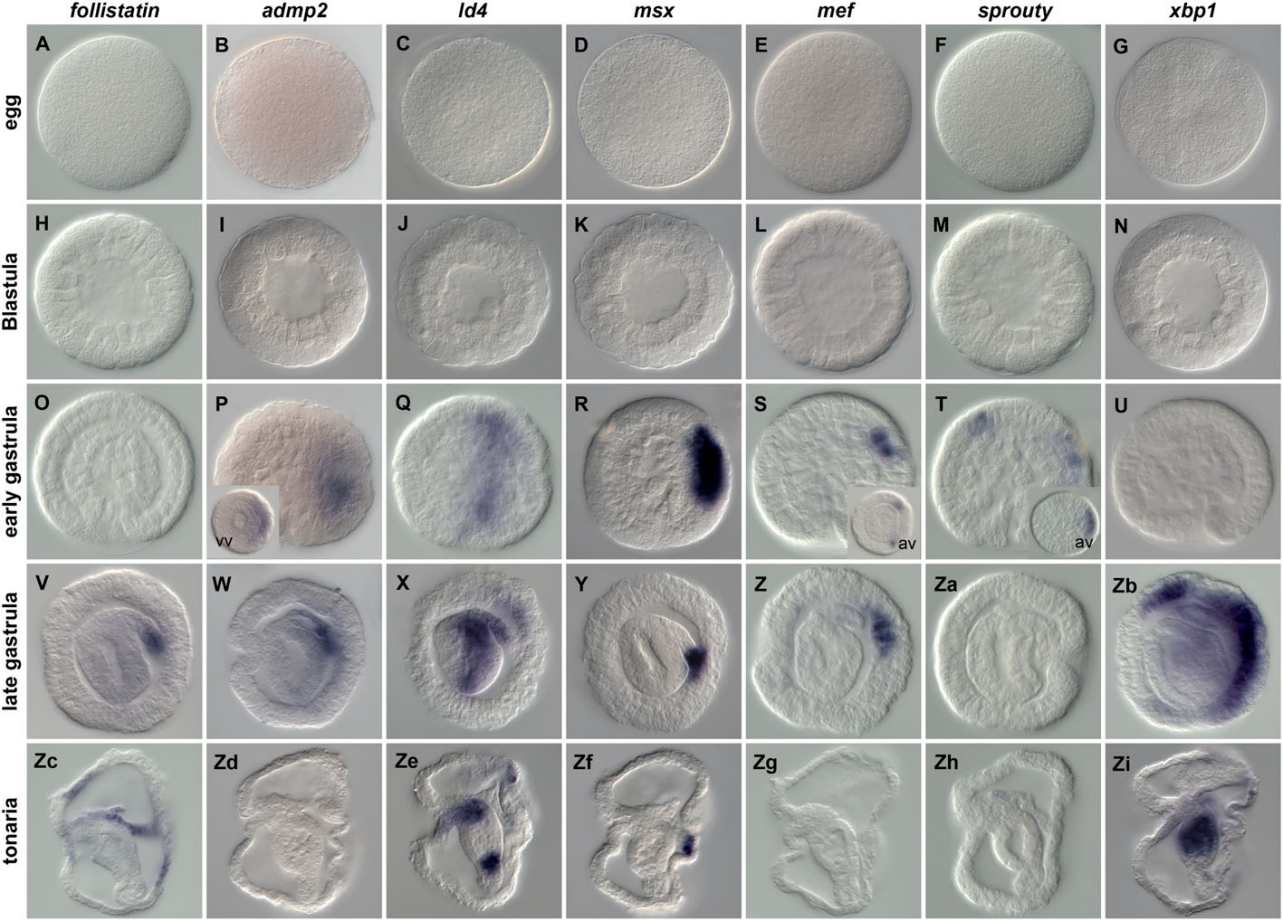
**Spatial expression of novel dorsal markers in *Ptychodera flava*.** Spatial distribution of *Pf-follistatin*, *Pf-admp2*, *Pf-id4*, *Pf-msx*, *Pf-mef*, *Pf-sprouty* and *Pf-xbp1* transcripts during normal development. Insert in P is vegetal view (vv) and in S and T are animal views (av).

**Fig. 3.**
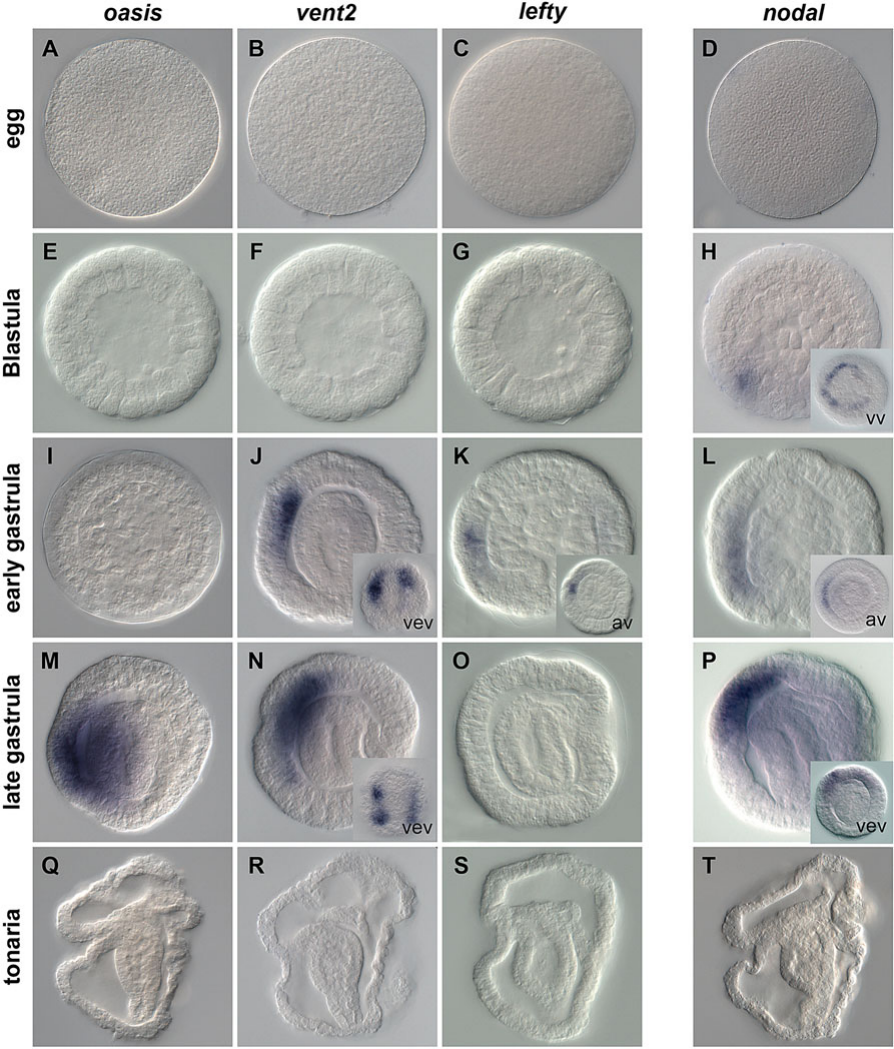
**Spatial expression of novel ventral markers in *Ptychodera flava*.** Spatial distribution of *Pf-oasis*, *Pf-vent2*, *Pf-lefty* and *Pf-nodal* transcripts during normal development. Insert in H is a vegetal view (vv), inserts in K and L are animal views (av) and inserts in J, N, P are ventral views (vev).

A recently performed RNAseq project has enabled us to identify a set of transcription factors and signaling molecules as well as to describe their spatial expression pattern during *P. flava* development (Table 1; Röttinger and Martindale, 2011). In order to extend the list of tissue and region-specific markers, we primarily focused on genes present in the transcriptome that have been described in the echinoderm ectoderm patterning GRN (Saudemont et al., 2010). Here we describe mRNA expression patterns of 18 previously uncharacterized genes during embryonic and larval development of *P. flava* (Figs 1-3). Phylogenetic analyses of the characterized transcriptional regulators, ligands and signaling modulators are shown in supplementary material Fig. S1.

We analyzed gene expression by *in situ* hybridization (WISH) and identified transcripts expressed specifically within the apical plate, the endoderm or the mesoderm (Fig. 1). The animal-vegetal axis is impossible to identify at pregastrula stages in *P. flava* so all early patterns are inferred to be in the same territories as those seen later. While there was no visible expression of transcripts in the egg (Fig. 1A-G), localized expression of *Pf-vent1* and *Pf-cripto* are detected asymmetrically as early as the blastula stage (Fig. 1H,I). Based on expression of both genes in the presumptive animal plate at gastrula stages (Fig. 1O,P) we suggest that the expression domain at earlier stages corresponds to the animal hemisphere. However, double WISH using a vegetal marker would be required to fully address this question. While expression of *Pf-vent1* starts to be reduced once the archenteron is formed (Fig. 1V,Zc), expression of *Pf-cripto* remains visible at late gastrula stage (Fig. 1W) before becoming undetectable at later stages (Fig. 1Zd). Expression of *Pf-sfrp1/5* in the presumptive apical domain is detected starting at the early gastrula stages and remains strongly expressed in the apical plate even after hatching in the tonaria larva (Fig. 1Q,X,Ze). Localized expression of *Pf-six3* in the anterior endoderm and presumptive apical plate becomes visible at the early gastrula stage (Fig. 1R) and remains detectable in the apical plate, anterior mesoderm and anterior portion of the mid-gut in later stages (Fig. 1Y,Zf). *Pf-tsg* transcripts are faintly detected in the animal region of early gastrula stages, but are clearly expressed in the apical plate, anterior mesoderm and anterior portion of the gut at the end of gastrulation (Fig. 1Z). After hatching, however, *Pf-tsg* is exclusively expressed in the mesoderm (protocoel, Fig. 1Zg). First expression of *Pf-hh* is only detected at the end of gastrulation in the apical plate (Fig. 1Za). In addition to its expression in the apical plate, *Pf-hh* transcripts are detected after hatching in the foregut (Fig. 1Zh), confirming previous observations (Arimoto, 2015). Finally, *Pf-irxA* is initially expressed in a region lying between the stomodeum and the apical domain at early gastrula stages (Fig. 1U) but is later expressed in the anterior endoderm (Fig. 1Zb) to be restricted only to the foregut and anterior midgut after hatching (Fig. 1Zi).

In the course of this WISH screen we also identified seven genes expressed exclusively within dorsal structures (Fig. 2). None of these seven genes are detected in eggs (Fig. 2A-G) or blastula stages (Fig. 2H-N). Expression of *Pf-admp2, Pf-id4, Pf-msx, Pf-mef* and *Pf-sprouty* (Fig. 2P-T) become visible in dorsal structures at the early gastrula stage. While *Pf-admp2* transcripts are detected in the dorsal ectoderm at the early gastrula stage (Fig. 2P), by the end of gastrulation its expression is restricted to the mesodermal part of the hydropore (the connection to the external environment, Fig. 2W) and not detected after hatching (Fig. 2Zd). *Pf-ld4* is expressed in the dorsal part of the invaginating gut as well as the apical most region of the dorsal ectoderm at the early gastrula stage (Fig. 2Q). At the end of gastrulation, *Pf-ld4* transcripts are localized within the same regions as well as in the mesoderm that has pinched of the archenteron (Fig. 2X). After hatching *Pf-ld4* is expressed in the foregut as well as the posterior region of the midgut (Fig. 2Ze). Initial expression of *Pf-msx* is detected in the dorsal ectoderm after the onset of gastrulation (Fig. 2R), and later in the dorsal most mesoderm (hydropore) (Fig. 2Y,Zf). *Pf-mef* expression is also initiated in the dorsal ectoderm at the early gastrula stage (Fig. 2S, however, in a smaller territory than *Pf-msx*), remains in the dorsal ectoderm at the end of gastrulation (Fig. 2Zg) and is undetectable in the tonaria larva (Fig. 2Zg). *Pf-sprouty* is expressed in the dorsal ectoderm as well as in an animal region between the stomodeum and the apical plate at the early gastrula (Fig. 2T). *Pf-xbp1* is expressed in the same domains later during development and visible only at the late gastrula stage (Fig. 2Zb). While *Pf-sprouty* is not detected after hatching (Fig. 2Zh), *Pf-xbp1* expression is restricted to the midgut in tonaria larvae (Fig. 2Zi).

We have also identified genes whose transcripts are restricted to the ventral ectoderm (Fig. 3). Expression of *Pf-oasis* is transient and only detected in the ventral ectoderm at the late gastrula stage (Fig. 3M). *Pf-vent2* expression is detected at the early and late gastrula stages in two distinct regions surrounding the stomodeum (Fig. 3J,N) while *Pf-lefty,* a potential modulator of Nodal signaling (Duboc et al., 2008), is expressed transiently in a small territory within the ectoderm at the early gastrula stage (Fig. 3K,O). Based on morphological signs at mid-late gastrula stage, we believe that *Pf-lefty* expression is localized to the ventral ectoderm (Fig. 3K, inset). However, double WISH using dorsal markers (i.e. *Pf-dlx*) is required to affirm this statement. None of the Pf-lefty transcripts were detected after hatching (Fig. 3Q-S).

We used RACE-PCR approaches to clone a full-length fragment of the *nodal* gene. Phylogenetic analysis (supplementary material Fig. S1) suggests the existence of at least one Nodal ligand in *P. flava*. Querying the recently released *P. flava* transcriptome database (http://molas.iis.sinica.edu.tw/hemichordate/; Chen et al., 2014) further supports the existence of only one Nodal in *P. flava* but needs to be confirmed once the *P. flava* genome (Freeman et al., 2012) will be released. The earliest expression of *Pf-nodal* was detected at the blastula (Fig. 3H) stage, in a ring of cells surrounding the future blastopore. During gastrula stages, blastoporal expression is no longer detected, however, *Pf-nodal* transcripts are refined to an ectodermal domain (Fig. 3L). At the late gastrula stage, just prior to hatching, *Pf-nodal* expression is detected in an ectodermal apico-ventral domain (Fig. 3P, inset) that appears to be slightly enriched towards the right side (Fig. 3P, inset). After hatching, no localized *Pf-nodal* expression is detected (Fig. 3T).

### Smad1/5 and Erk1/2 signaling are activated in dorsal territories

**Fig. 4.**
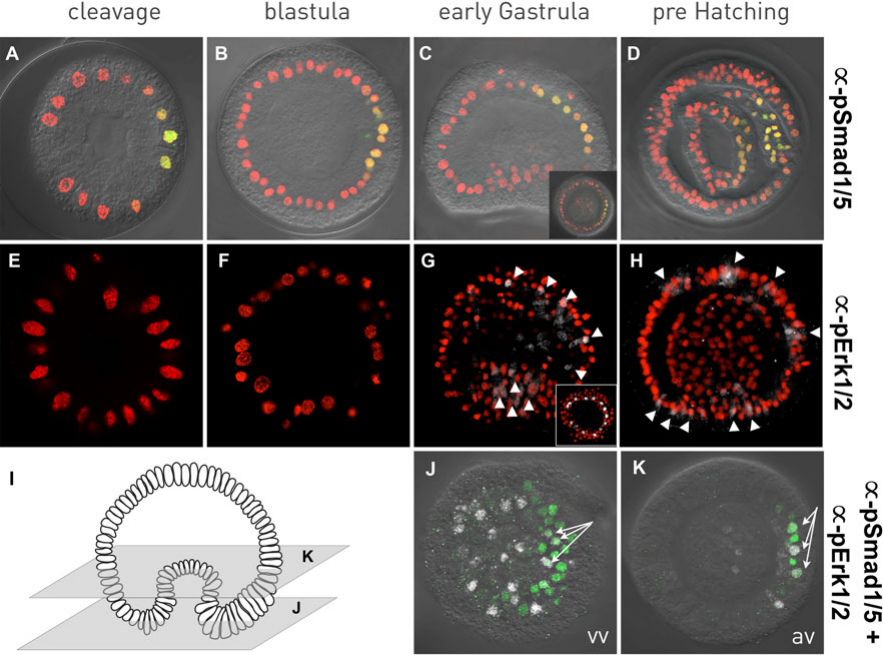
**pSmad1/2 and pERK1/2 activation pattern during normal *P. flava* development.** (A-D) Spatial distribution of phospho-Smad1/5 (green) positive cells in relation to Hoechst (nuclei in red) counterstaining. Insert in C is an animal view. (E-H) Spatial distribution of phospho-Erk1/2 (white) positive cells in relation to Hoechst (nuclei in red) counterstaining. G and H are stacks of four Z-acquisitions to better show the activation profile of pERK (white arrowheads). Insert in G is an animal view. (I) Schematic representation of an early gastrula stage indicating the focal planes represented in J and K that show the spatial distribution of pSmad1/5 and pErk1/2 simultaneously (vv, vegetal view; av, animal view). White arrows indicate cells in which pSmad1/5 and pErk1/2 are detected in the same nuclei.

In order to visualize activity of Nodal signaling, we performed IHC using 8 different commercial antibodies made against mouse phospho-Smad2 /Smad3. However, none of these reagents allowed us to detect a clear localization/activation of Pf-Smad2/3 despite attempts to optimize fixation, blocking, and antibody dilutions (data not shown). Bmp2/4 is a transcriptional target of Nodal signaling in echinoderm larvae (Duboc et al., 2004) and required to determine dorsal fates in echinoderm (Angerer et al., 2000; Duboc et al., 2004) and hemichordate (Lowe et al., 2006) embryos. Therefore, we analyzed the localization of pSmad1/5 using a monoclonal antibody against the phosphorylated form of Smad1/5. This reagent allowed us to detect a localized signal as early as the late cleavage and early blastula stages (Fig. 4A,B). Based on the expression patterns of Bmp2/4 and the dorsal expression of its potential downstream targets [*Pf-smad6* (Röttinger and Martindale, 2011), *Pf-admp2*, *Pf-msx* (Fig. 2T,R)], it appears that early activation of pSmad1/5 occurs in presumptive dorsal ectodermal cells. However, a ventral marker, to perform double IHC is required to confirm this statement. Activity of Smad1/5 remains asymmetric during gastrulation movements (Fig. 4C) and is clearly present in cells of the dorsal endo-, meso-, and ectoderm (Fig. 4D), giving support to the hypothesis that the early expression is also on the dorsal side.

In chordates FGF signaling is required for endoderm and mesoderm formation (Bertrand et al., 2011; Imai et al., 2002; Kim et al., 2000; Slack et al., 1989) as well as dorsoventral (DV) patterning (Fürthauer, 2004). In order to observe the activation pattern of this pathway we analyzed the spatial activation of Erk phosphorylation during *P. flava* development using a monoclonal antibody that recognizes an activated form of Erk1/2 (Fig. 4E-H, pErk1/2, Cell Signaling) (Yung et al., 1997). No signal was detected during early embryonic development in *P. flava*, however, after the onset of gastrulation, activated Erk1/2 is detected in individual cells surrounding the blastopore, in the center of the vegetal plate, as well as in the dorsal ectoderm (Fig. 4G). At the end of gastrulation, prior to hatching, staining persists in individual cells surrounding the blastopore, the apical region and the dorsal ectoderm (Fig. 4H).

In order to analyze whether either Bmp2/4/pSmad1/5 or Fgf/pErk, or Bmp2/4/pSmad1/5 and Fgf/pErk, pathways are activated in a given cell we stained fixed embryos simultaneously with antibodies against pSmad1/5 and pErk1/2 (Fig.4I-K). While pErk1/2 positive cells are detected throughout the vegetal plate, some dorsally located cells also display positive pSmad1/5 signal (Fig. 4I,J). In an optical section that is located closer to the animal pole, one can still observe pErk1/2 positive cells in the invaginating endomesoderm. However, one can also observe that pErk/2 and pSmad1/5 staining is exclusively located to the dorsal region of the ectoderm (Fig. 4I,K). Thus, our analysis shows that all three scenarios can be detected; in any one given cell we detect pErk, or pSmad1/5, or signals showing that both pathways can be activated in the same cell (Fig. 4K).

### Partially overlapping roles of Nodal, Bmp2/4 and Erk1/2 signaling during *P. flava* development

In order to assess the roles of these signaling pathways during *P. flava* development, we performed recombinant protein as well as pharmacological drug treatments previously used in echinoderms (Duboc et al., 2005; Röttinger et al., 2004; Saudemont et al., 2010) and/or hemichordates (Green et al., 2013; Lowe et al., 2006). To perturb Nodal signaling, we treated embryos with mouse Nodal (mNodal) and human Nodal (hNodal), mLefty and the Alk4/5/7-Receptor inhibitor SB431542. To activate Bmp2/4 signaling, we used zebrafish Bmp (zBmp4) and mBmp4 and to inhibit Erk1/2 signaling we used the Mek inhibitor U0126. We performed dose-response experiments using various concentrations during *P. flava* development to determine the optimal concentrations for each of the components (supplementary material Fig. S2). hNodal, mLefty and mBmp2, did not induce any observable phenotype at any concentration (data not shown) and were not pursued.

**Fig. 5.**
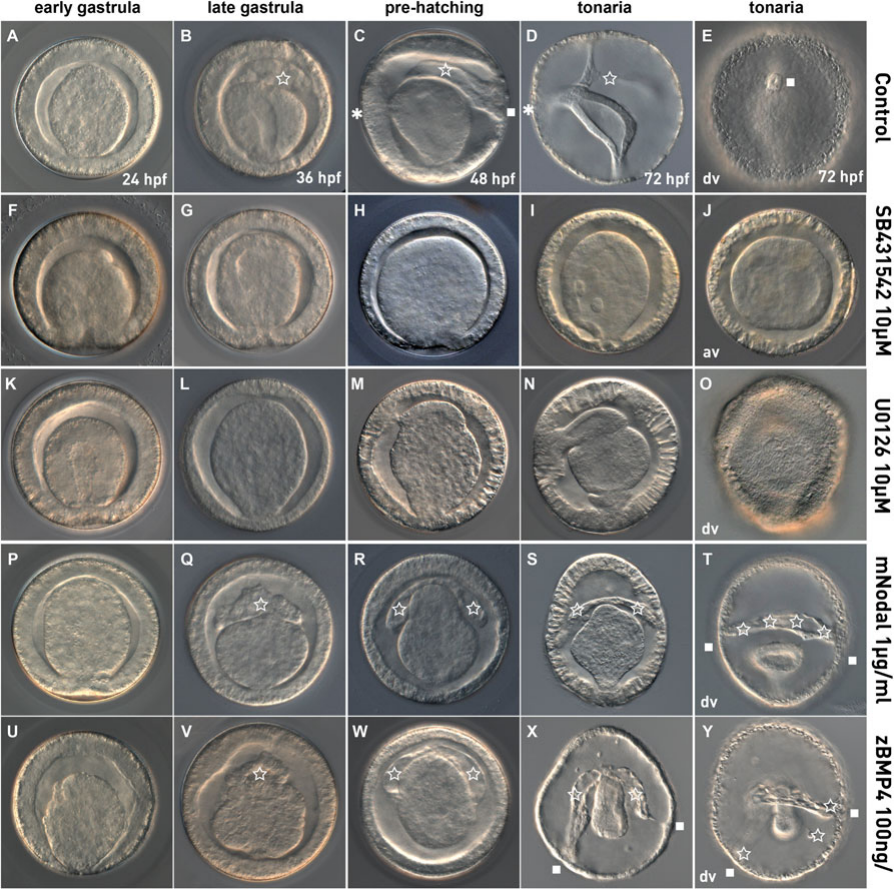
**Phenotypes observed after SB431542, U0126, mNodal and zBmp4 treatments.** Control embryos (A-E) or SB4315432 (F-J), U0126 (K-O) mNodal (P-T) and zBmp4 (U-Y) treated embryos. All images are lateral views, except E, O, T and Y that are dorsal views (dv) and J, which is an animal view (av). The asterisk indicates the mouth (ventral), the star the protocoel (mesoderm) and the square the hydropore (dorsal).

Inhibition of Alk4/5/7 by SB431542 starting at the 2-cell stage caused severe phenotypes affecting mesoderm formation and dorsoventral patterning (Fig. 5F-J). At the end of gastrulation when the protocoel forms in control embryos (Fig. 5B), mesodermal structures are neither detected at that stage in SB431542-treated embryos nor at later stages after hatching (Fig. 5G-J). Not only is mesoderm formation affected by inhibition of Alk4/5/7 signaling but also the formation of ventral and dorsal structures as indicated by the absence of the mouth and the hydropore (Fig. 5H-J). In order to determine the period during which *P. flava* embryos are sensitive to SB431542, we performed pharmacological treatments starting at various periods of time during embryonic development (supplementary material Fig. S3B-G). Treatments from 4–12 hours post fertilization (hpf) or treatments starting at 12 hpf have no visible effects (supplementary material Fig. S3E-G). However, extended treatments from 4–24 hpf (supplementary material Fig. S3B,C) or 4–36 hpf (not shown) block mesoderm formation and dorsoventral patterning. These results indicate that Nodal signaling is required early during development and that only a continuous disruption for at least 20 h affects proper mesoderm formation and D/V pattering in *P. flava*.

Similar to our results with SB431542, blocking the transduction of Mek/Erk1/2 signaling by U0126 completely prevents mesoderm formation at gastrula and later stages (Fig. 5L-O). While the mouth forms simultaneously in control and U0126-treated embryos, the latter fail to form the protocoel and the hydropore opening in the dorsal ectoderm (Fig. 5M-O). Inhibition of this pathway at various periods of time and that start at different moments of development, show that all U0126 treatments starting prior to 24 hpf block mesoderm and hydropore formation (supplementary material Fig. S3H-K), indicating that functional Mek/Erk1/2 signaling during the first 24 h after fertilization is crucial for *P. flava* development. The phenotypes observed in both pharmacological inhibition treatments suggest a functional connection of the Nodal/Alk4 and Mek/Erk1/2 pathways in mesoderm formation and potentially in patterning the dorsoventral axis.

In order to analyze the effects of Nodal and/or Bmp2/4 over-activation in *P. flava*, we treated zygotes with recombinant zBmp4 or mNodal respectively. Surprisingly, both treatments caused the same phenotypes, reflected by a severe dorsalization of the treated embryos (Fig. 5P-T,U-Y). This dorsalization becomes clearly visible just prior to hatching when in control embryos the protocoel extends towards the dorsal ectoderm and fuses with the latter to form the hydropore (Fig. 5C). In treated embryos, however, the protocoel extends towards the ectoderm not in a single spot, but either in a broad region forming a slit like hydropore that extends to more than half of the larval circumference (Fig. 5S,T), or in several regions to form multiple hydropores (Fig. 5X,Y). In addition, hydropore formation in mNodal or zBmp4 treated embryos is slightly delayed compared to control animals (Fig. 5C,R,W). Our analysis to define the zBmp or mNodal activation sensitive period shows i) that all zBmp4 treatments starting prior to 36hpf cause the formation of an excess of mesoderm (supplementary material Fig. S3R-U), ii) only the treatment starting after 24 hpf induces the formation of ectopic hydropores (supplementary material Fig. S3V) and iii) that all mNodal treatments staring prior to 36 hpf cause dorsalization of the ectoderm (supplementary material Fig. S3M-P).

### Inhibition of Mek and Nodal signaling affects pERK signaling but not dorsal activation of pSmad1/5

**Fig. 6.**
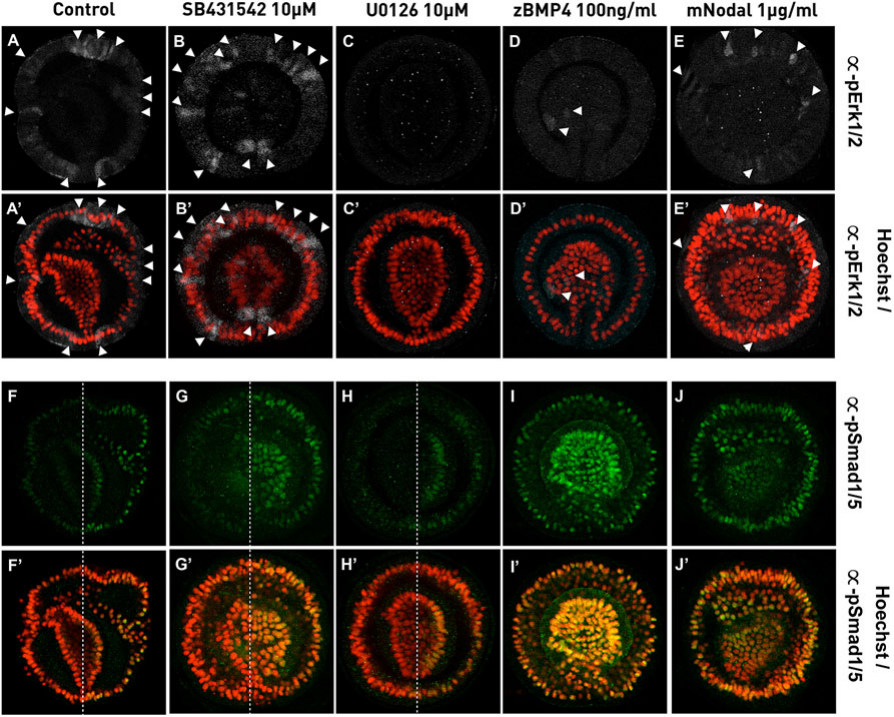
**Analysis of pErk1/2 and pSmad1/5 activity after drug and recombinant protein treatments.** Control embryos (A,A′,F,F′) and SB4315432 (B,B′,G,G′), U0126 (C,C′,H,H′) zBmp4 (D,D′,I,I′) or mNodal (E,E′,J,J′) treated embryos. (A–E) pErk1/2 staining alone or (A′–E′) counterstained with Hoechst to visualize DNA/nuclei. (F–J) pSmad1/5 staining alone or (F′–J′) counterstained with Hoechst to visualize DNA/nuclei. All images are pre-hatching larva oriented as described in Fig. 1. White arrowheads in (A,A′–E,E′) indicate the position of pErk1/2 positive cells. Dashed lines in (F′,F–H,H′) indicate the limit between the larva ventral and dorsal sides.

To gain a better understanding of the observed phenotypes on ERK signaling and Bmp/24 signaling, we performed whole mount immunocytochemistry using the antibodies described above (Fig. 4). In control embryos, at the end of gastrulation, pErk staining is detected in individual cells of the apical region, surrounding the blastopore and within the dorsal ectoderm (Figs 4H, 6A). As expected, inhibition of Mek using U0126 entirely blocks Erk signaling in treated embryos (Fig. 6C). In a similar manner, zBmp4 treatments also strongly reduce the activity of Erk signaling (Fig. 6D). Clear effects on Erk signaling are also observed in embryos in which we perturbed Nodal signaling using SB431542 or mNodal. While we observed an increase of pErk positive cells using SB431542 (Fig. 6B), Erk signaling appears slightly decreased in mNodal treated embryos (Fig. 6E). However, in both treatments the characteristic asymmetric dorsoventral distribution of pErk positive cells (Figs 4H, 6A) is disrupted and these cells are evenly distributed throughout the animal ectoderm (Fig. 6B,E). This observation is consistent with the radialization and dorsalization phenotypes observed in SB431542 (Fig. 5F-J) and mNodal (Fig. 5P-T) treatments, respectively.

In late gastrula stages, pSmad1/5 staining is detected asymmetrically in cells of the dorsal endo-, meso-, and ectoderm (Figs 4C, 6F). Unsurprisingly, pSmad1/5 positive cells are detected throughout the entire embryos in zBmp4 or mNodal treatments (Fig. 6I,J) which is in agreement with the dorsalizing phenotypes observed when those recombinant proteins are ectopically applied to the culture (Fig. 5P-T,U-Y). Somewhat unexpectedly though, U0126 treatments that block hydropore formation, have no visible effect on asymmetric pSmad1/5 activation (Fig. 5K-O). An even more surprising observation is that SB431542-treated embryos that lack any morphological signs of dorsoventral patterning (Fig. 5F-J) retain the asymmetric distribution of pSmad1/5 positive cells in dorsal structures (Fig. 6G) compared to control embryos (Fig. 6F).

### Inhibition of Alk4/5/7 blocks expression of ventral and mesodermal markers and expands the apical territory

**Fig. 7.**
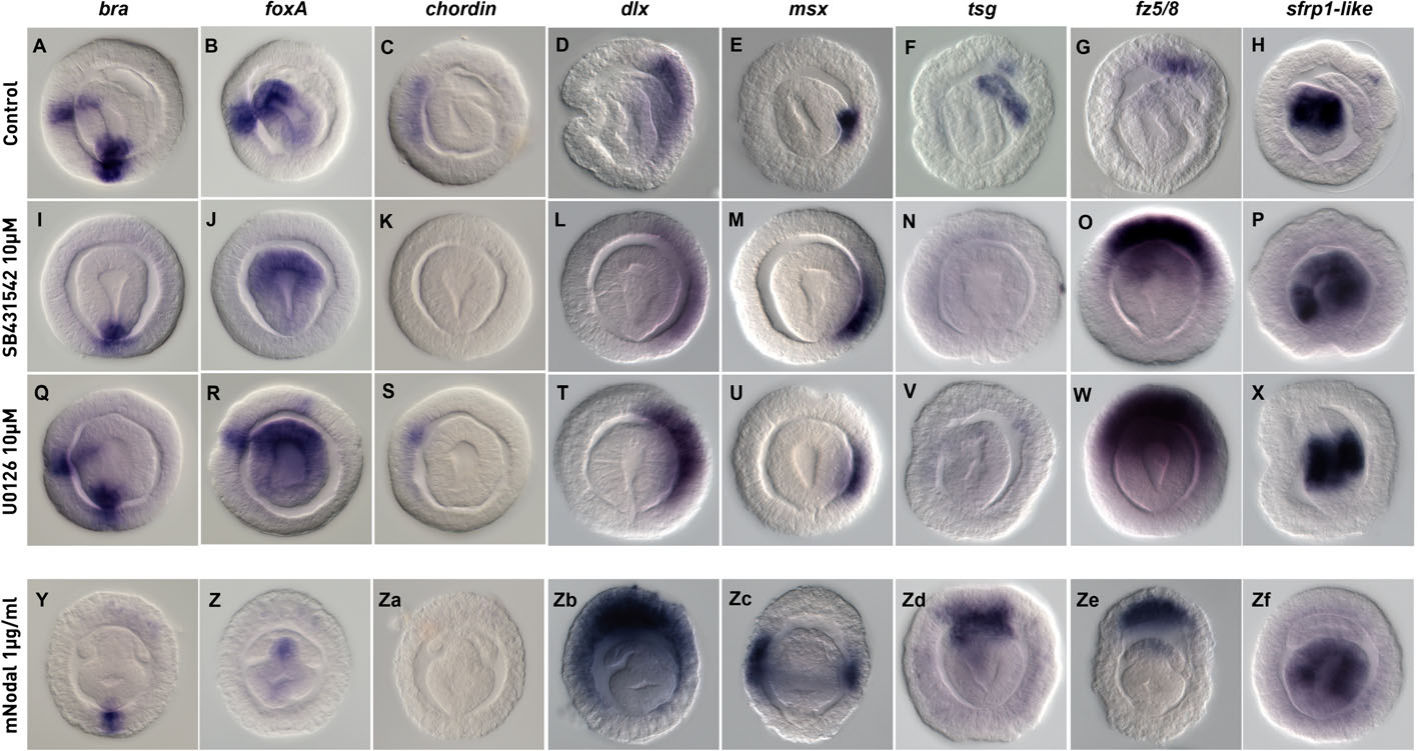
**Effects of SB431542, U0126 and mNodal on specification of ectoderm, mesoderm and endoderm.** WISH performed at the late gastrula/pre-hatching stage of control embryos (A–and SB431542 (I–P), U0126 (Q–X) or mNodal (Y–Zf) treated embryos. The effects of these treatments on the gene expression program of the ectoderm, mesoderm of endoderm were analyzed by WISH with the indicated probes.

To further characterize the molecular effects of the above described phenotypes, we performed WISH on SB431542, U0126 and mNodal-treated embryos (Fig. 7). We used antisense probes that recognize endogenous transcripts of *Pf-bra, Pf-foxA* and *Pf-chordin* expressed in the ventral ectoderm (Fig. 7A-C), *Pf-dlx* and *Pf-msx* expressed in the dorsal ectoderm or hydropore respectively (Fig. 7D,E) as well as *Pf-tsg*, *Pf-fz5/8* and *Pf-sfrp1-like* whose transcripts are localized to the endoderm, mesoderm or apical domain, respectively (Fig. 7F-H). For technical reasons associated with the restricted spawning behavior of *P. flava* this analysis does not include zBmp4-treated embryos.

In embryos in which Alk4/5/7 function is impaired using SB431542 the formation of the protocoel (mesoderm), the mouth (ventral) and the hydropore (dorsal) is impaired (Fig. 5F-J). In agreement with this phenotype, we observed inhibition of the ventral and mesodermal expression *Pf-bra, Pf-foxA, Pf-chordin* and *Pf-tsg* (Fig. 7I-K,N) in SB431542-treated embryos. Surprisingly, but consistent with results shown in Fig. 6G, expression of the dorsal markers *Pf-dlx* and *Pf-msx* was not downregulated but instead slightly expanded by this treatment (Fig. 7L,M). However, it is noticeable that even in the absence of the protocoel (that also expresses *Pf-msx,* Fig. 7E), *Pf-msx* expression is detected in the dorsal ectoderm (Fig. 7M). While SB431542 treatments have no effect on endodermal expression of *Pf-sfrp1-like* (Fig. 7P), we observed a clear expansion of the *Pf-fz5/8* expression domain reflecting a potential extension of the apical domain (Fig. 7O).

Inhibition of Mek/Erk signaling using U0126 affects formation of the protocoel as well as the hydropore in the dorsal ectoderm (Fig. 5K-O). In agreement with this observation, no expression of the mesodermal marker *Pf-tsg* is detected in U0126-treated embryos (Fig. 7V). Consistent with our observations in SB431542-treated embryos, expression domains of the dorsal markers *Pf-dlx* and *Pf-msx* are slightly larger compared to control embryos (Fig. 7T,U), while in U0126 treatments the territory of *Pf-fz5/8* within the apical domain is expanded (Fig. 7W). Expression of the ventral and endodermal markers *Pf-bra, Pf-foxA, Pf-chordin* and *Pf-sfrp1-like* remain unaffected by U0126 (Fig. 7Q-S,X).

Applying an exogenous source of recombinant mNodal protein causes the dorsalization of the embryos as reflected by the formation of multiple hydropores and the absence of a mouth (Fig. 5U–Y). The expression of *Pf-bra*, *Pf-foxA* and *Pf-chordin* in the ventral ectoderm is abolished (Fig. 7Y,Z,Za), while *Pf-dlx* and *Pf-msx* expression is detected in the entire circumference of the dorsalized larvae (Fig. 7Zb,Zc) which is consistent with this phenotype. Expression of *Pf-tsg* in the mesoderm and *Pf-sfrp1-like* in the endoderm remains unaffected (Fig. 7Zd,Zf), while Pf-fz5/8 expression in the apical domain appears slightly increased (Fig. 7Ze).

## DISCUSSION

In this study we analyzed the roles of the MAPK/Erk, Nodal and Bmp2/4 signaling pathways in the indirect developing hemichordate *P. flava*. Although we attempted for several spawning seasons to microinject mRNA encoding *nodal* or *bmp2/4* as well as morpholinos targeted against these transcripts, the survival rate of injected oocytes was not sufficient to test individual gene function. Thus, optimization of an mRNA or morpholino delivery system for *P. flava* is crucially needed. For the present study, we were restricted to pathway perturbation experiments using pharmacological inhibitors and recombinant proteins. Nonetheless, several lines of evidence enable us to propose that the Mek/Erk pathway is required for mesoderm formation, Bmp2/4 is required for dorsal cell fate specification and that Nodal signaling is involved in mesoderm formation, ventral cell fate specification and potentially A/P patterning.

### Ectopic Bmp2/4 signaling induces dorsalization of *P. flava*

Previous studies have shown that *Pf-bmp2/4* and its potential downstream target *Pf-dlx* are expressed in dorsal territories (Harada et al., 2001; Harada et al., 2002; Röttinger and Martindale, 2011). In the current study, we show that Bmp signaling is already asymmetrically activated during cleavage stages and later within dorsal territories of all three germ layers further suggesting that Bmp2/4 signaling is involved in patterning dorsal fates in *P. flava*. In agreement with this idea, treatments with recombinant zBmp4, radializes the activation of pSmad1/5 and induces the formation of ectopic hydropores that normally form only on the dorsal side. Interestingly, functional studies in the direct developing hemichordate *S. kowalevskii*, echinoderms, and our results in *P. flava* show that regardless of the source of *bmp2/4* expression, the activity of the Bmp pathway in all studied ambulacrarians is always confined to the dorsal ectoderm where it is responsible for the specification of dorsal fates (Duboc et al., 2004; Lapraz et al., 2009b; Lowe et al., 2006). Our attempts to inhibit Bmp signaling using dorsomorphin (Hao et al., 2008; Yu et al., 2008) or Noggin (Bayramov et al., 2011) did not affect either the development of *P. flava*, nor caused any noticeable changes of pSmad1/5 activation (data not shown). Nonetheless, taken together our above-described results strongly suggest that Bmp signaling is involved in patterning dorsal structures during *P. flava* development.

### Erk signaling is required for mesoderm and hydropore formation

Activation of Erk signaling takes place in individual cells that form a ring in the vegetal hemisphere prior to and after the onset of gastrulation. At the end of gastrulation pErk positive cells are detected in the blastoporal region, the apical domain and throughout the dorsal ectoderm. The latter activation pattern is similar to *Pf-sprouty, Pf-xpb1* and *Pf-pea* expression (Röttinger and Martindale, 2011), suggesting a functional link between these factors and Erk1/2 signaling in *P. flava*. We also showed that inhibition of Mek/Erk signaling prevents the formation of the mesodermally derived protocoel that is confirmed by the loss of *Pf-tsg* expression in U0126-treated embryos. Mek/Erk signaling has been shown to be crucial for mesoderm induction and/or specification in vertebrates (Kimelman, 2006), arthropods (Muha and Müller, 2013), direct developing hemichordates (Green et al., 2013) and echinoderms (Fernandez-Serra et al., 2004; Röttinger et al., 2004). Our data strengthen the idea that the role of MAPK/Erk signaling in mesoderm formation is a shared bilaterian feature.

Interestingly, the hydropore is also absent in U0126-treated embryos suggesting that pErk signaling may be involved in specifying dorsal fates in *P. flava*. However, pSmad1/5 positive cells in the dorsal ectoderm as well as expression of the dorsal markers *Pf-dlx* and *Pf-msx* are not affected by U0126. Thus, pErk in the dorsal ectoderm could be required for attracting the dorsal mesoderm towards the ectoderm. The importance of an inductive interaction between the mesoderm and dorsal ectoderm for hydropore formation is supported by the observation that pErk alone in the ectoderm is not sufficient to induce hydropore formation. In fact, SB4351542-treated embryos do not form either mesoderm or the hydropore even though pErk activity is expanded.

### Multiple roles of Nodal signaling in hemichordates

In *P. flava*, we have identified one single *nodal* gene that is expressed in a ring of cells within the vegetal hemisphere prior to the onset of gastrulation and in the ventral ectoderm during gastrulation. While vertebrates possess multiple copies of Nodal (Loose and Patient, 2004), the direct and indirect developing echinoderms that have been studied to date posses only one *nodal* gene that is expressed exclusively within ventral territories throughout embryonic development (Duboc et al., 2004; Smith et al., 2008). It is important to note that while in all three ambulacrarian species studied, *nodal* is expressed within the ventral ectoderm, but in *P. flava*, *nodal* transcripts are also detected within the vegetal pole prior to gastrulation. This suggests a divergent transcriptional control of this gene and potential additional functions in hemichordates.

To study the role of Nodal signaling in *P. flava*, we performed pharmacological drug treatments using SB431542 that inhibits the function of Alk4/5/7, the TGF-β receptor activated by Nodal, Activin or TGF-β itself. Hence, one could argue that SB431542 treatments could affect signaling other than the one mediated by Nodal. While this may be true, we have not been able to identify Activin by degenerate PCR or in our RNAseq datasets, and TGF-β appears to be expressed well after the end of gastrulation prior to hatching (data not shown). Therefore, we currently believe that the phenotypes observed with SB431542 treatments in *P. flava* are caused by inhibition of Nodal signaling during embryonic development.

### Nodal signaling is required for mesoderm formation and specifying ventral fates

Inhibition of Nodal/Alk4/5/7 signaling in *P. flava* prevents the formation of the mesoderm that is confirmed by the absence of *Pf-tsg* expression in treated animals. Interestingly, we observe *Pf-nodal* expression at the beginning of gastrulation in a ring of cells surrounding the future blastopore. While those cells are not the presumptive mesodermal cells (Henry et al., 2001), it is interesting to note that pErk positive cells, whose inhibition blocks mesoderm formation, are detected in a similar pattern. Therefore, our current results suggest that similar to what has been described in vertebrates (Mathieu et al., 2004), Nodal and Erk signaling may interact to induce/maintain the formation of the mesoderm/protocoel in *P. flava.* Further gene-specific epistasis experiments are, however, required to understand if both pathways interact directly, interdependently or in parallel.

**Fig. 8.**
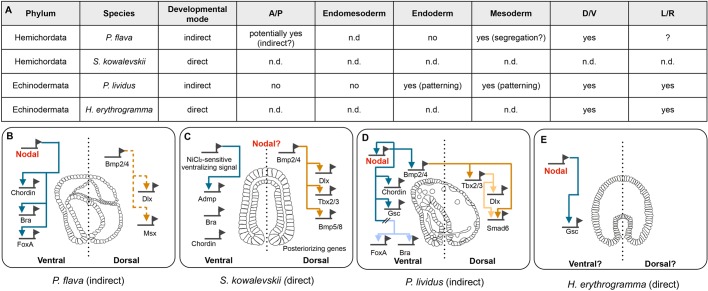
**Comparison of the role and molecular mechnaisms of Nodal signaling in ambulacrarians.** (A) Identified roles of Nodal signaling in ambulacrarians. (B-E) diagrams representing the molecular mechanism underlying D/V patterning in ambulacrarians. Hemichordates: *P. flava* (indirect development) and *S. kowalevskii* (direct development) (this study; Lowe et al., 2006)*.* Echinoderms: *P. lividus* (indirect development) and *H. erythrogramma* (direct development) (Duboc et al., 2004; Lapraz et al., 2009b; Saudemont et al., 2010; Smith et al., 2008; Su et al., 2009).

Inhibition of Nodal or Alk4/5/7 also severely affects the dorsoventral axis as SB431542-treated embryos lack their ventral mouth and the dorsal hydropore. While our gene expression analysis confirms the loss of ventral markers, neither pSmad1/5 staining in dorsal territories, nor the expression of the dorsal markers *Pf-dlx* and *Pf-msx* were affected by this treatment. This observation strengthens the idea that the mesodermally derived protocoel generates a hydropore-inducing cue responsible for the formation of this dorsal opening in *P. flava*. In addition, this is particularly interesting because it suggests that the Nodal and Bmp2/4 signaling pathways are functionally uncoupled in hemichordates compared to echinoderms (Fig. 8). In fact, in echinoderms ventral *nodal* expression is responsible for inducing ventral expression of *bmp2/4* which in turn diffuses to the dorsal ectoderm where it induces expression of its downstream targets (Duboc et al., 2004; Lapraz et al., 2009b; Saudemont et al., 2010; Su and Davidson, 2009). In contrast, hemichordate *bmp2/4* is expressed and active exclusively in dorsal structures where it is required to specify dorsal fates (this study; Harada et al., 2002; Lowe et al., 2006; Röttinger and Martindale, 2011).

Although the dorsalizing phenotype we obtain by treating *P. flava* embryos with recombinant mNodal protein is tantalizing, it is also puzzling and unexpected because *Pf-nodal* is expressed in the ventral ectoderm and has known ventralizing effect in echinoderms (Duboc et al., 2004). In addition, this mNodal treatment induces ubiquitous activation of pSmad1/5 and radialization of the dorsal markers *Pf-dlx* and *Pf-msx*, suggesting that the observed phenotype is the result of activation of the Bmp2/4 pathway. Treatments with recombinant Activin A, or the optimization of the microinjection protocol in *P. flava* for the overexpression of *Pf-nodal* are required to confirm our current observations.

### A potential role for Nodal signaling in A/P patterning

Inhibition of Nodal signaling induces the extension of the apical domain marker *Pf-fz5/8* that could reflect an anteriorization of the treated embryo, the first demonstration of this role in any ambulacrarian. While additional apical markers are required to confirm this observation, this extension could be simply due to the absence of a putative signal emitted by the mesodermally derived protocoel that restricts *Pf-fz5/8* expression to the apical domain. However, it is noticeable that *Pf-cripto*, an essential co-receptor for Nodal signaling (Gritsman et al., 1999) in vertebrates, is expressed in the apical domain from blastula to late gastrula stages. This expression pattern suggests a role for Nodal signaling in patterning the A/P axis or, perhaps the larval nervous system in *P. flava*. The *Pf-cripto* expression pattern also suggests that the mesodermal and D/V patterning phenotypes we observed in SB431542-treated embryos may be linked to Cripto-independent Nodal signaling as has been described during early vertebrate embryogenesis (Liguori et al., 2008). Alternatively, there could also be additional Nodal co-receptors that we have not been able to identify in our RNAseq datasets.

Recent studies in the cnidarian *Hydra*, the gastropod mollusc *Lottia gigantea* and echinoderms have suggested an ancestral function of Nodal signaling in establishing axial asymmetries (Watanabe et al., 2014) and left-right asymmetries (Bessodes et al., 2012; Duboc et al., 2005; Grande and Patel, 2009). The first morphological sign of L/R asymmetry in *P. flava* is detected in juveniles in which the hydropore is connected to the external environment on the left side of the midline (Dawydoff, 1948). With the exception of a slight enrichment of *Pf-nodal* expression within the right ventral ectoderm at late gastrula stages, we could not determine any further indications for a potential role of Nodal signaling in establishing L/R asymmetries during larval stages in *P. flava*. In addition, the transcription factor Pitx, a conserved downstream target of Nodal signaling during the establishment of L/R asymmetries in metazoans (Boorman and Shimeld, 2002; Duboc et al., 2005; Grande and Patel, 2009) is expressed within the ventral and dorsal midlines of the juveniles (supplementary material Fig. S4). A more thorough functional analysis, in regard to the positioning of the juvenile hydropore is required to fully address this question.

Taken together, the current data suggest that the role of Nodal signaling in patterning the D/V axis is a shared feature among ambulacrarians while this pathway has been co-opted in hemichordates for mesoderm formation (and potentially for patterning the A/P axis) prior to the divergence of the chordate lineage. It would be important to understand the role of Nodal signaling in a direct developing hemichordate for a thorough and complete comparison of the role of Nodal in early development and adult body plan formation in this group of animals.

## MATERIAL & METHODS

### Animal care and treatments

Adult *P. flava* were collected from Paiko lagoon on the leeward shore of Oahu, Hawaii. Animal culture and induction of spawning were previously described in (Röttinger and Martindale, 2011; Tagawa et al., 1998b). Treatments with the indicated reagents were carried out by exposing embryos to various concentrations at various time intervals. All reagents were renewed every 12 h to maintain the concentrations and the activity of pharmacological inhibitors or recombinant proteins. Per treatment several hundreds to thousands of embryos from a mix of males and females were used. Treatments were repeated several times during one spawning season as well as over the course of several spawning seasons. If not indicated otherwise, representative phenotypes observed in at least 80% of the treated embryos are shown. All animal experiments carried out conformed to the relevant regulatory standards. The reagents used were: mNodal (#1315-ND), hNodal (#3218-ND), mLefty (#994-LF), zBmp4 (#1128-BM), mBmp4 (#5020-BP) and hNoggin (#6057-NG) from R&D Systems, Inc; SB431542 (#S4317), Dorsomorphin (#P5499) and U0126 (Sigma, #U120) from Sigma Aldrich Co, LLC.

### Cloning *P. flava* genes

With the exception of *Pf-nodal*, all sequences described in this study were isolated in the course of a transcriptome analysis described in (Röttinger and Martindale, 2011). Sequences were used to sub-clone full-length open reading frames (ORF, or the longest possible ORF) of the genes of interest into pGemT® (Promega Corp.) from mixed stage cDNA. If needed we performed 5′ and 3′ RACE PCR on mixed stage RACE cDNA using the SMART^TM^ RACE cDNA Amplification Kit (Clonetch Inc.). Degenerate, RACE-PCR as well as full-length primers used to clone *Pf-nodal* are indicated in supplementary material Table S1. Nucleotide sequences have been submitted to GenBank and the accession numbers are indicated in Table 1.

**Table 1.**
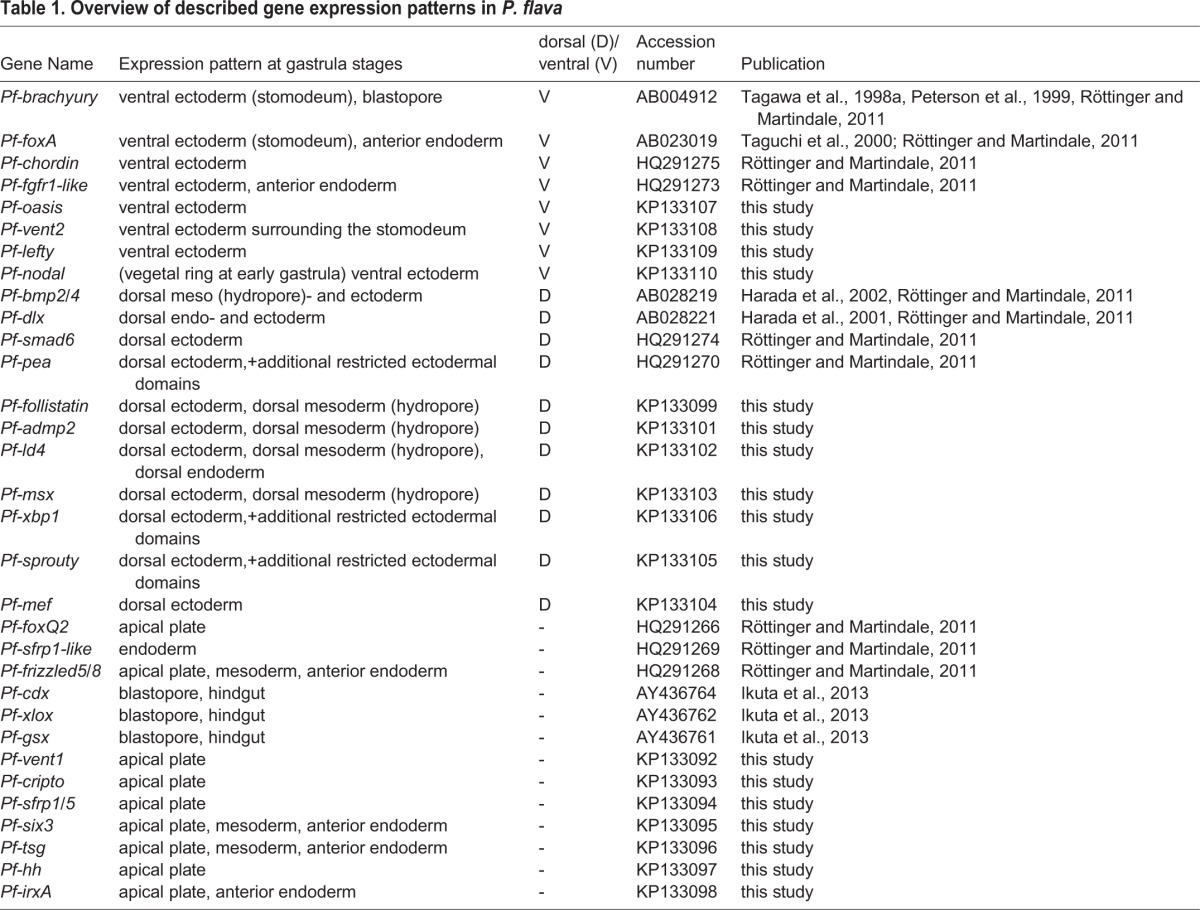
**Overview of described gene expression patterns in *P. flava***

### Phylogenetic analysis

Sequences from the following animals were gathered from NCBI protein database (http://www.ncbi.nlm.nih.gov/protein) and are abbreviated as follows: **Am** – *Alligator mississippiensis*; **Amil** – *Acropora millepora*; **Bb** – *Branchiostoma belcheri*; **Bj** – *Branchiostoma belcheri japonicas*; **Bf** – *Branchiostoma floridae*; **Cf** – *Crepidula fornica*; **Cg** – *Crassostrea gigas*; **Ci** – *Ciona intestinalis*; **Ct** – *Capitella teleta*; **Dm** – *Drosophila melanogaster*; **Dr** – *Danio rerio*; **Gg** – *Gallus gallus*; **Hp** – *Hemicentrotus pulcherrimus*; **Hs** – *Homo sapiens*; **Lg** – *Lottia gigantea*; **Ls** – *Lymnaea stagnalis*; **Mm** – *Mus musculus*; **Nv** – *Nematostella vectensis*; **Oh** – *Ophiophagus hannah*; **Pf** – *Ptychodera flava*; **Pl** – *Paracentrotus lividus*; **Pm** - *Petromyzon marinus*; **Pv** – *Patella vulgata*; **Sk** – *Saccoglossus kowalevskii*; **Sp** – *Strongylocentrotus purpuratus*; **Tc** – *Tribolium castaneum*; **Tg** – *Tegillarca granosa*; **Xl** – Xenopus laevis; **Xt** – *Xenopus tropicalis*.

Sequences were aligned using MUSCLE (http://www.ebi.ac.uk/Tools/msa/muscle/) and then trimmed according the conserved protein domains. Protein domain boundaries were identified using the SMART protein prediction database (http://smart.embl-heidelberg.de/). Phylogenetic trees were constructed using MrBayes (Huelsenbeck and Ronquist, 2001; Ronquist and Huelsenbeck, 2003) and are based upon five million generations using a “mixed” evolutionary model. Trees were imported and edited using FigTree (version 1.4.0, http://tree.bio.ed.ac.uk/software/figtree) and finalized using Adobe Illustrator (CS6).

### WISH and immunohistochemistry

The protocol for digoxigenin-labeled antisense RNA probe synthesis (Megascript, Ambion, Inc), embryo fixation and WISH has been described in (Röttinger and Martindale, 2011). Animals for immunohistochemistry were fixed were fixed and permeabilized in 0.1 M HEPES (pH6.9), 50 mM EGTA, 10 mM MgSO_4_, 0.5 M Maltose, 4% paraformaldehyde (Methanol+RNase free/Electron Microscopy Science) and 0.2% Triton X-100 for 2 h at room temperature, followed by washing in PBS/Triton X-100 0.2% (Amiel and Houliston, 2009).

pSmad1/5 and pErk1/2 were visualized by incubation the given antibodies diluted at 1:200 in blocking solution [PBT+10% normal goat serum (NGS)] overnight at 4°C. Following six washes in PBT, embryos were incubated with the secondary antibody (anti-mouse Ig for pErk1/2, anti-rabbit Ig for pSmad1/5, both diluted at 1:250) and with Hoechst dye 33258 (1 μg/ml) included to stain DNA for at least 4 h to overnight at 4°C on a shaking rocker. PBS was used for washes between antibodies. Specimens were mounted in 80% glycerol and imaged using a Zeiss LSM 710 confocal microscope running the LSM ZEN software (Carl Zeiss).

Antibodies tested and used (all Cell Signaling Technologies, Inc): Phospho-Smad1 (Ser463/465) / Smad5 (Ser463/465) / Smad8 (Ser426/428), #9511S; Phospho-Smad2 (Ser463/465) / Smad3 (Ser423/425), #9510S; Phospho-Smad2 (Ser465/467), #3101S; Phospho-Smad2 (Ser245/250/255), #3104S; Phospho-Smad2 (Ser465/467), #3108S; Phospho-Smad2 (Ser465/467), #3108P; Phospho-Smad3 (Ser423/425), #9520S; Smad3, #9513; Smad3, #9523S.

All expression and activation pattern can be consulted at: http://www.kahikai.org/index.php?content=genes.

## Supplementary Material

Supplementary Material
