# Supplementary material for: Nodal signaling is required for mesodermal and ventral but not for dorsal fates in the indirect developing hemichordate, Ptychodera flava
Source: Biol Open. 2015 May 15;4(7):830–42. doi: 10.1242/bio.011809 (PMC4571091; doi:10.1242/bio.011809)
Supplement: Supplementary Material [file supp_4_7_830__index.html]

Nodal signaling is required for mesodermal and ventral but not for dorsal fates in the indirect developing hemichordate, Ptychodera flava — Nodal signaling is required for mesodermal and ventral but not for dorsal fates in the indirect developing hemichordate, Ptychodera flava — Supplementary Material 

# Nodal signaling is required for mesodermal and ventral but not for dorsal fates in the indirect developing hemichordate, *Ptychodera flava*

## BIO011809 Supplementary Material

- Supplementary Material
