# Supplementary material for: Nodal signaling is required for mesodermal and ventral but not for dorsal fates in the indirect developing hemichordate, Ptychodera flava
Source: Biol Open. 2015 May 15;4(7):830–42. doi: 10.1242/bio.011809 (PMC4571091; doi:10.1242/bio.011809)
Supplement: Supplementary Material [file supp_bio.011809_BIO011809supp.pdf]

Phylogenetic trees

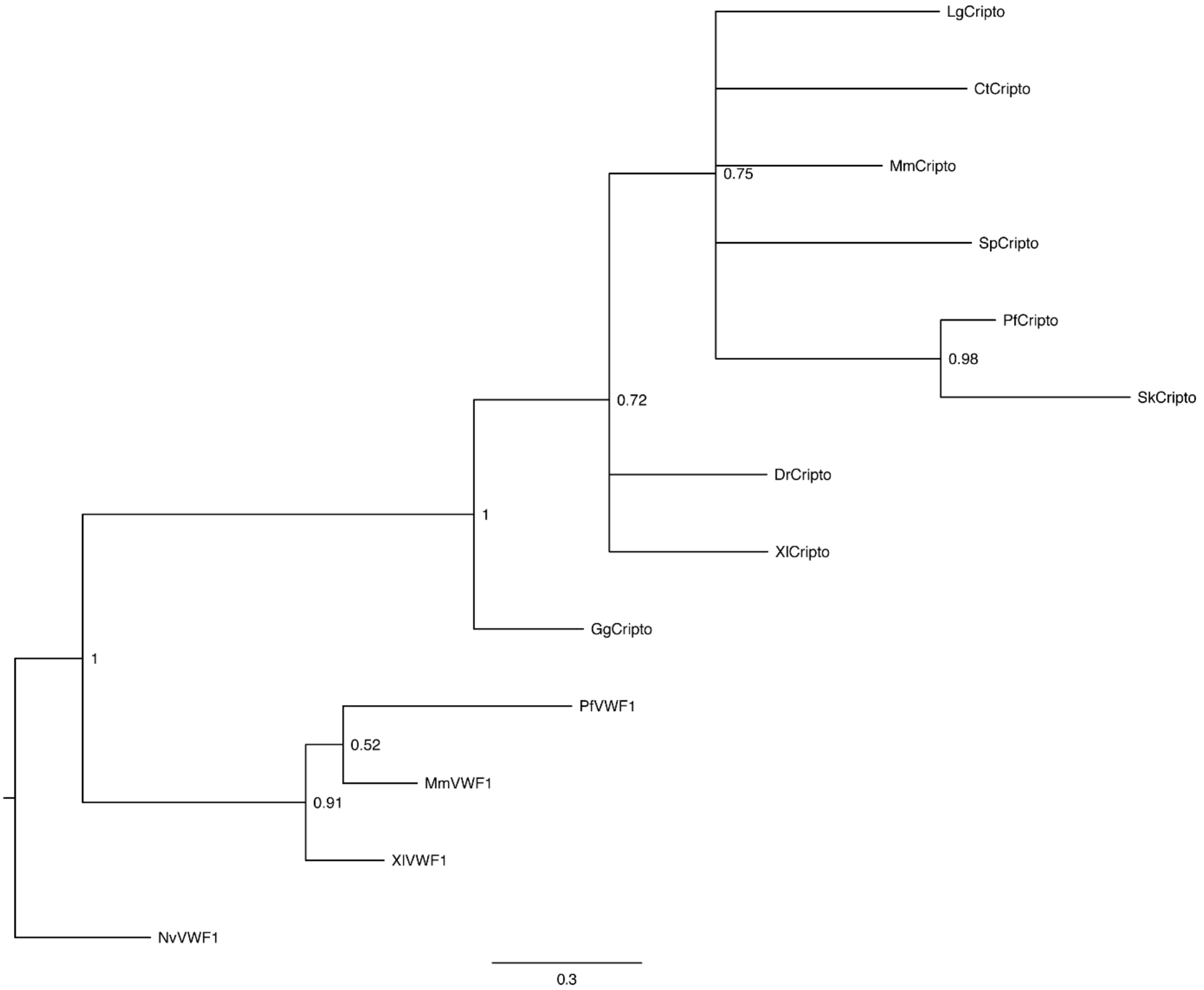

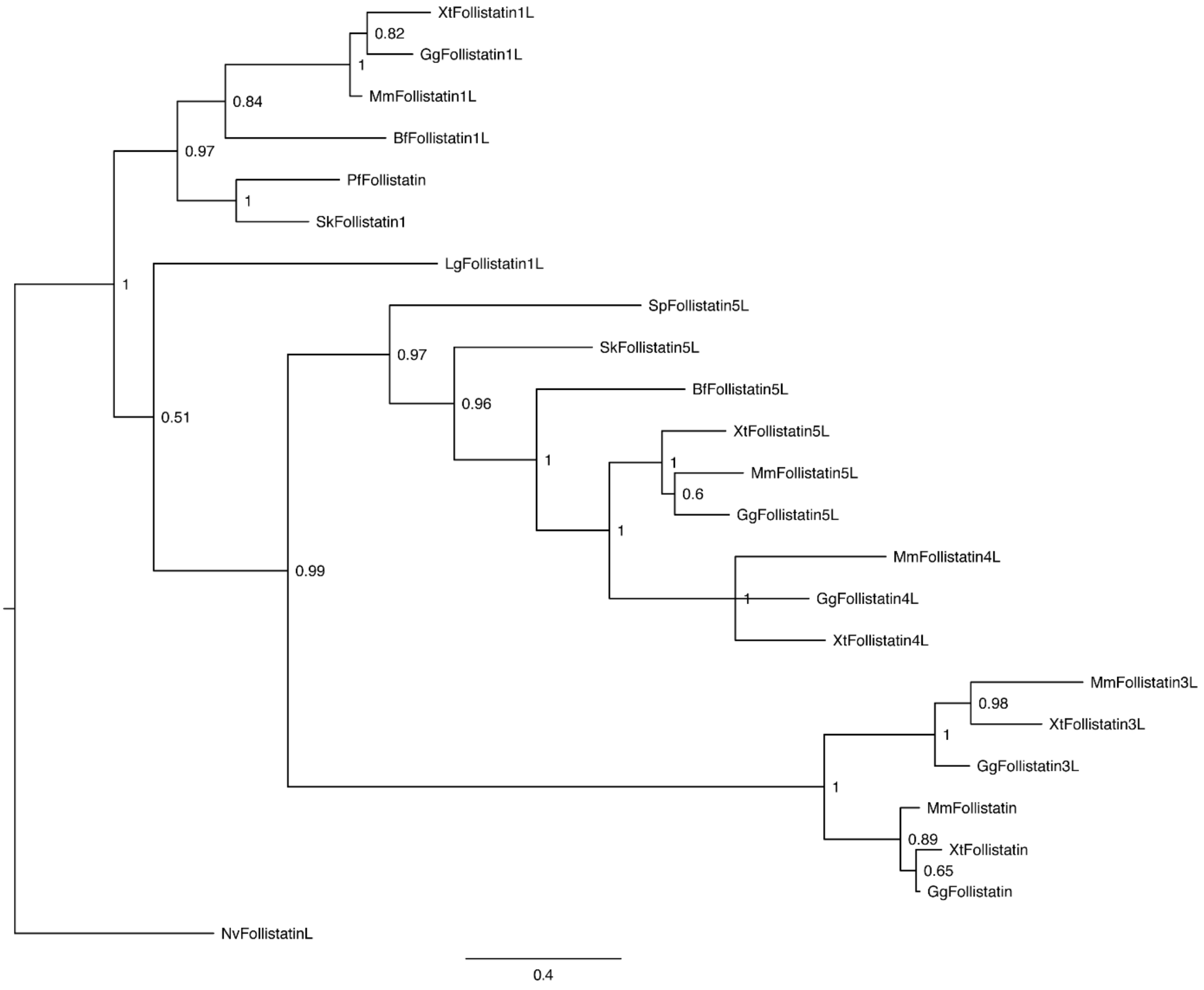
XtFollistatin1L
0.82
GgFollistatin1L
1
MmFollistatin1L
0.84
BfFollistatin1L
0.97
PfFollistatin
1
SkFollistatin1
LgFollistatin1L
1
SpFollistatin5L
0.97
SkFollistatin5L
0.96
BfFollistatin5L
0.51
XtFollistatin5L
1
1
MmFollistatin5L
0.6
GgFollistatin5L
1
MmFollistatin4L
1
GgFollistatin4L
0.99
XtFollistatin4L
MmFollistatin3L
0.98
XtFollistatin3L
1
GgFollistatin3L
1
MmFollistatin
0.89
XtFollistatin
0.65
GgFollistatin
NvFollistatinL
0.4

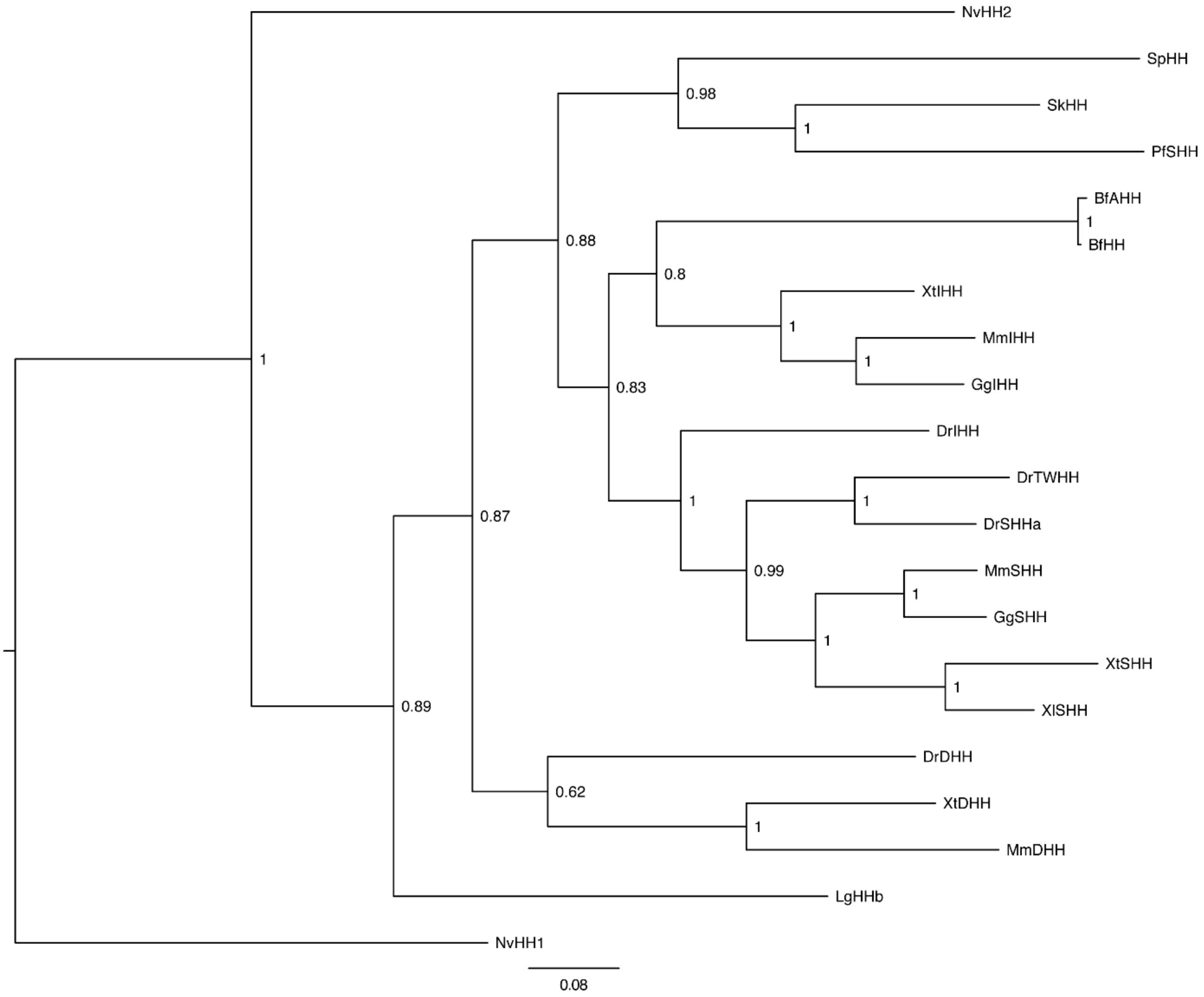
NvHH2
SpHH
0.98
SkHH
1
PfSHH
BfAHH
1
BfHH
0.88
0.8
XtIHH
1
MmIHH
1
GgIHH
1
0.83
DrIHH
DrTWHH
1
1
DrSHHa
0.87
0.99
MmSHH
1
GgSHH
1
XtSHH
1
XlSHH
0.89
DrDHH
0.62
XtDHH
1
MmDHH
LgHHb
NvHH1
0.08

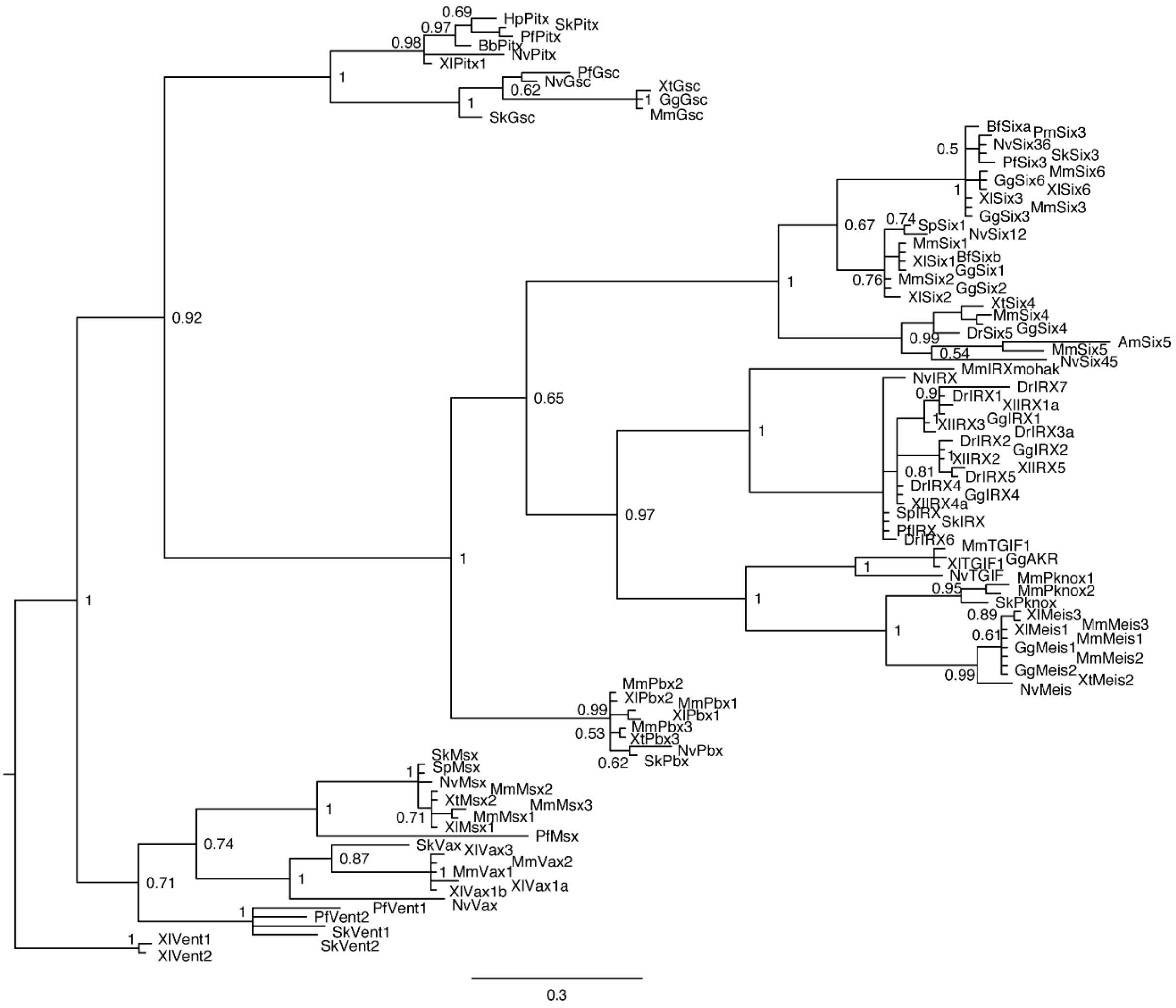

HpPitx
SkPitx
PfPitx
BbPitx
NvPitx
XlPitx1
PfGsc
NvGsc
XtGsc
GgGsc
MmGsc
SkGsc
BfSixa
PmSix3
NvSix36
SkSix3
PfSix3
MmSix6
GgSix6
XlSix6
XlSix3
MmSix3
GgSix3
SpSix1
NvSix12
MmSix1
XlSix1
BfSixb
GgSix1
MmSix2
GgSix2
XlSix2
XtSix4
MmSix4
DrSix5
GgSix4
AmSix5
MmSix5
NvSix45
MmIRXmohak
NvIRX
DrIRX7
DrIRX1
XlIRX1a
XlIRX3
GgIRX1
DrIRX3a
DrIRX2
GgIRX2
XlIRX2
DrIRX5
XlIRX5
DrIRX4
GgIRX4
XlIRX4a
SpIRX
SkIRX
PfIRX
DrIRX6
MmTGIF1
XlTGIF1
GgAKR
NvTGIF
MmPknox1
MmPknox2
SkPknox
XlMeis3
MmMeis3
XlMeis1
MmMeis1
GgMeis1
MmMeis2
GgMeis2
XtMeis2
NvMeis
MmPbx2
XlPbx2
MmPbx1
XlPbx1
MmPbx3
XtPbx3
NvPbx
SkPbx
SkMsx
SpMsx
NvMsx
MmMsx2
XtMsx2
MmMsx3
MmMsx1
XlMsx1
PfMsx
SkVax
XlVax3
MmVax2
MmVax1
XlVax1b
XlVax1a
NvVax
PfVent1
PfVent2
SkVent1
SkVent2
XlVent1
XlVent2
0.3

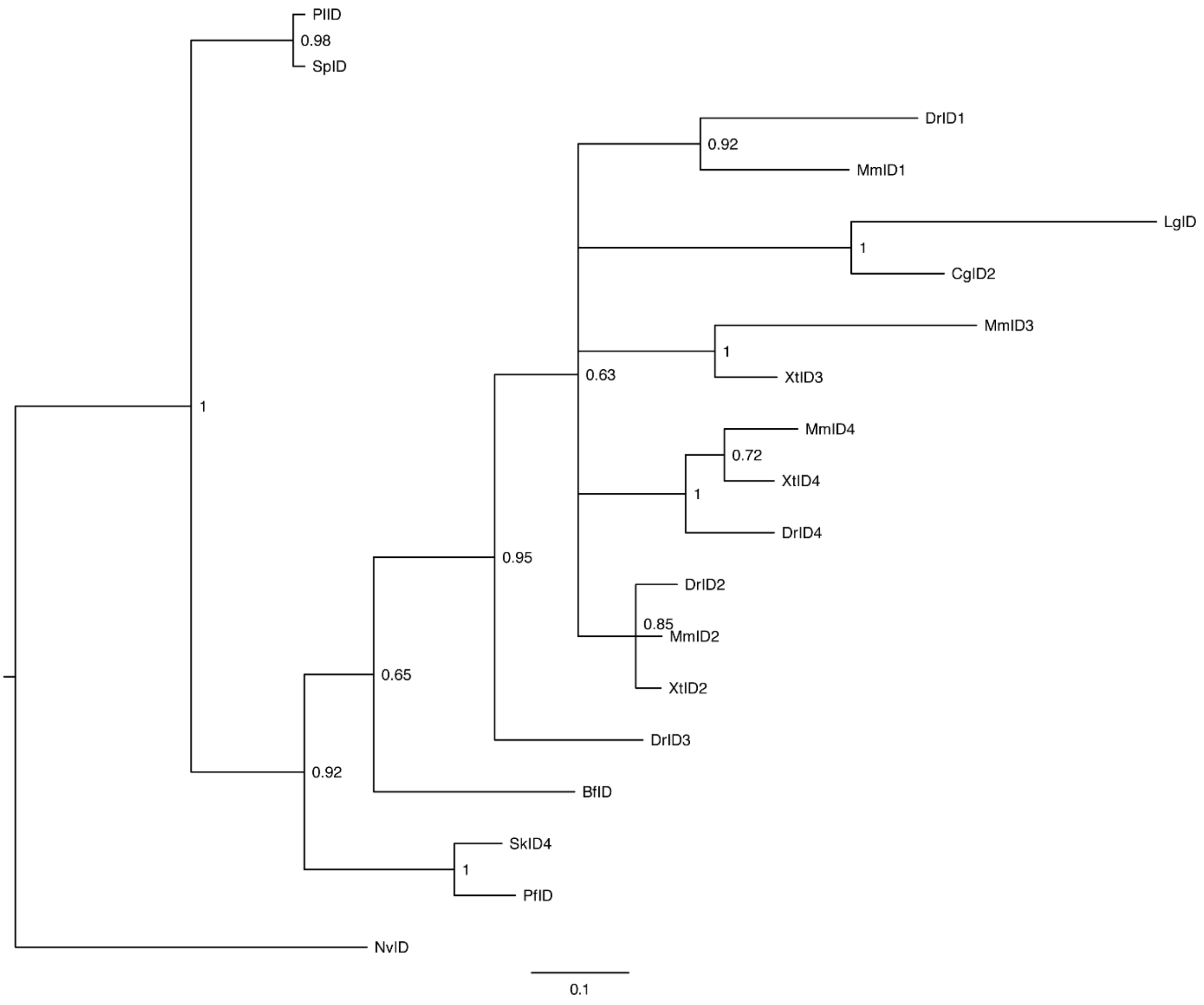
PlID
0.98
SpID
DrID1
0.92
MmID1
LgID
1
CgID2
MmID3
1
XtID3
1
0.63
MmID4
0.72
XtID4
1
DrID4
0.95
DrID2
0.85
MmID2
XtID2
0.65
DrID3
0.92
BfID
SkID4
1
PfID
NvID
0.1

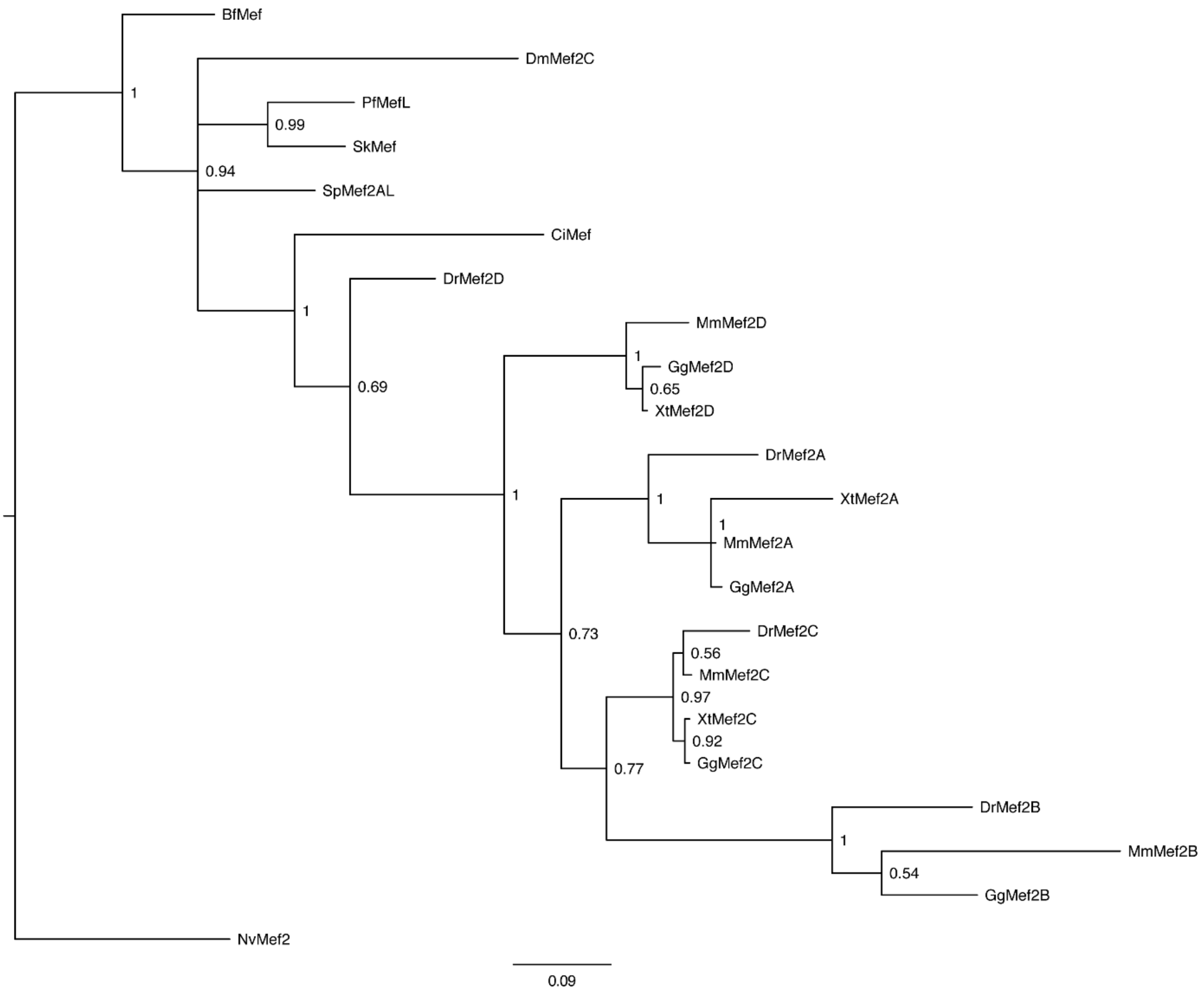
BfMef
1
DmMef2C
PfMefL
0.99
SkMef
0.94
SpMef2AL
CiMef
1
DrMef2D
0.69
MmMef2D
1
GgMef2D
0.65
XtMef2D
1
DrMef2A
1
XtMef2A
1
MmMef2A
GgMef2A
0.73
DrMef2C
0.56
MmMef2C
0.97
XtMef2C
0.92
GgMef2C
0.77
DrMef2B
1
MmMef2B
0.54
GgMef2B
NvMef2
0.09

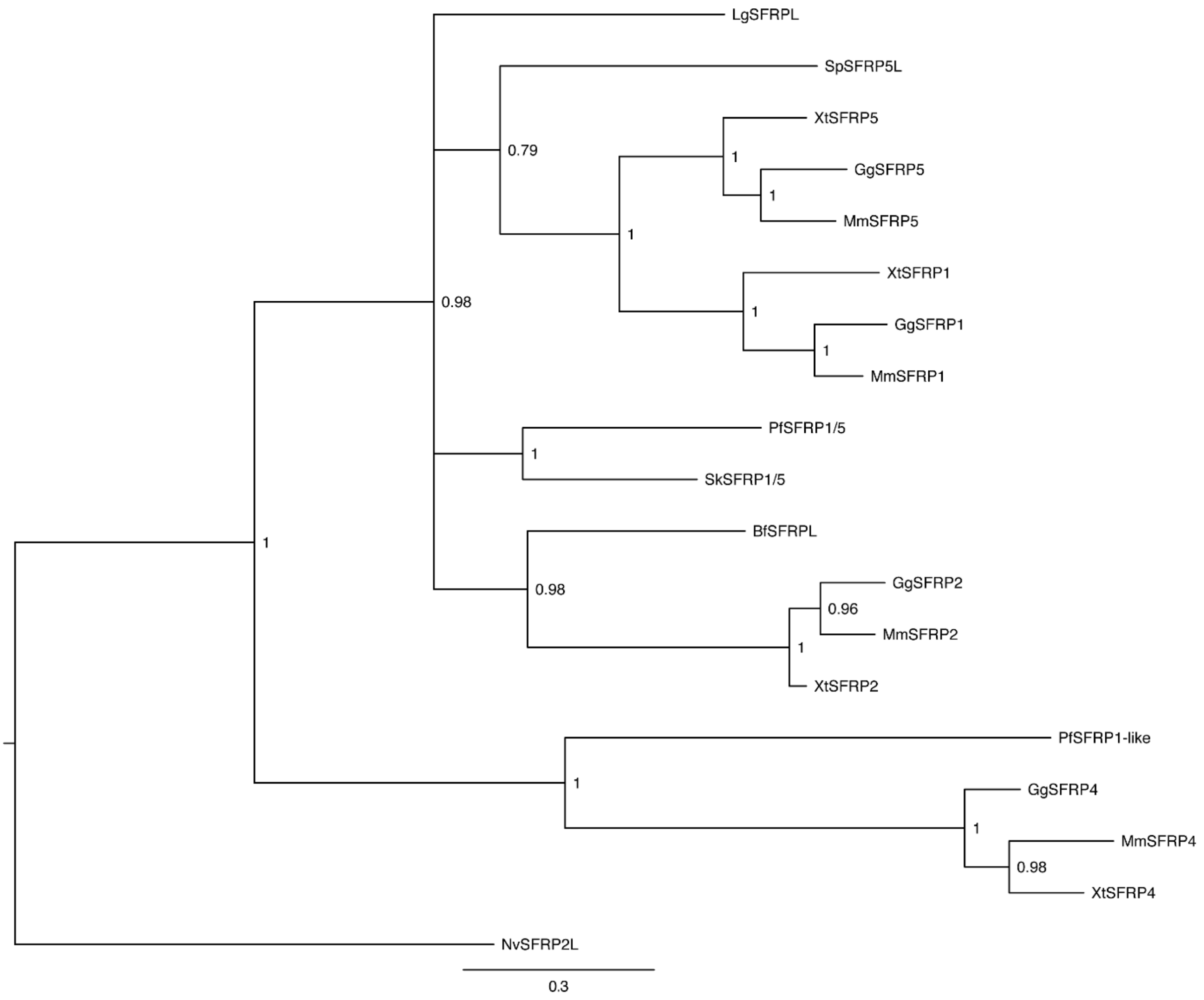
LgSFRPL
SpSFRP5L
XtSFRP5
GgSFRP5
MmSFRP5
XtSFRP1
GgSFRP1
MmSFRP1
PfSFRP1/5
SkSFRP1/5
BfSFRPL
GgSFRP2
MmSFRP2
XtSFRP2
PfSFRP1-like
GgSFRP4
MmSFRP4
XtSFRP4
NvSFRP2L
0.79
0.98
0.98
0.96
0.98
0.3

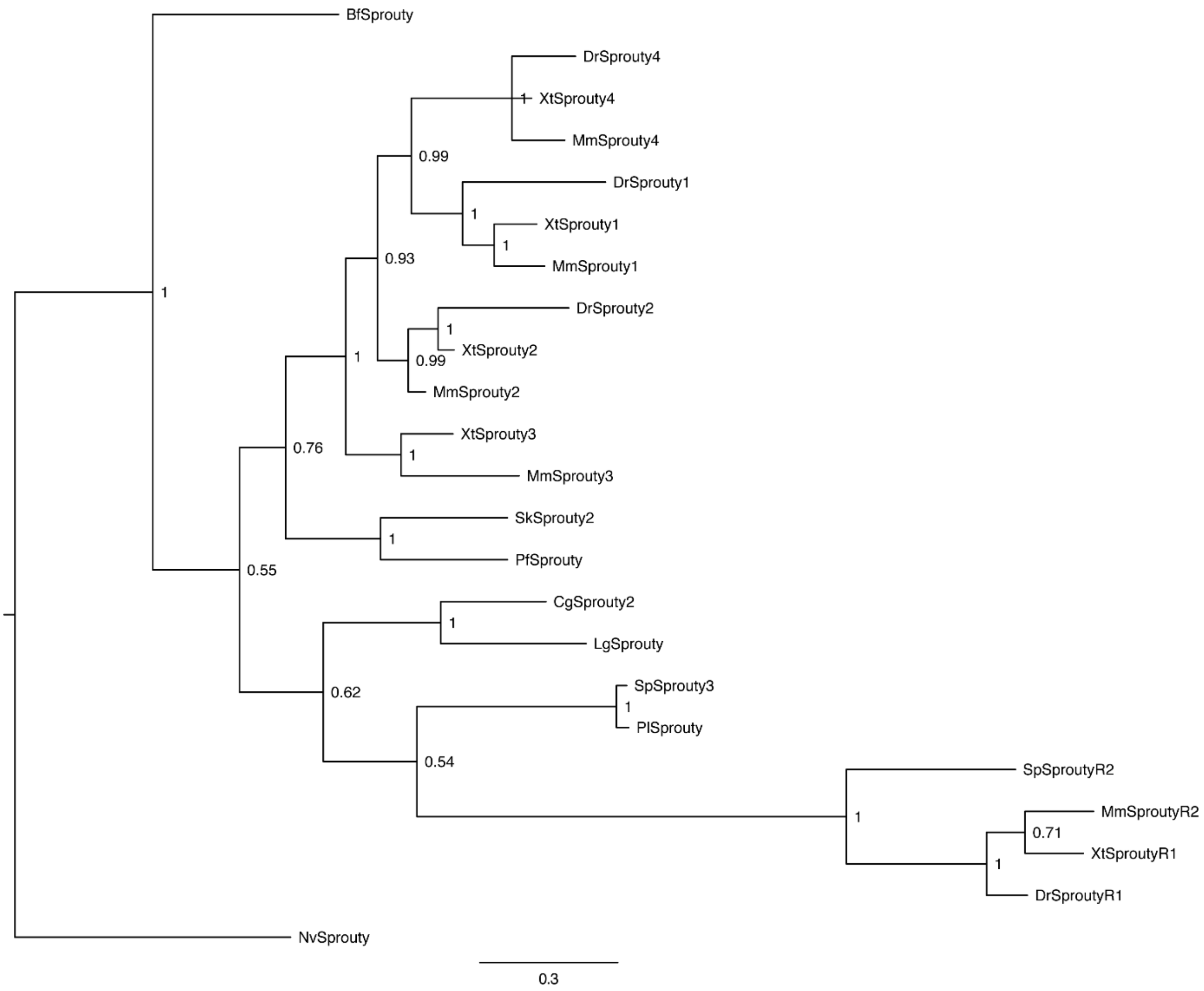
BfSprouty
DrSprouty4
XtSprouty4
MmSprouty4
0.99
DrSprouty1
1
XtSprouty1
1
MmSprouty1
0.93
DrSprouty2
1
XtSprouty2
0.99
MmSprouty2
1
1
0.76
XtSprouty3
1
MmSprouty3
SkSprouty2
1
PfSprouty
0.55
CgSprouty2
1
LgSprouty
0.62
SpSprouty3
1
PlSprouty
0.54
SpSproutyR2
1
MmSproutyR2
0.71
XtSproutyR1
1
DrSproutyR1
NvSprouty
0.3

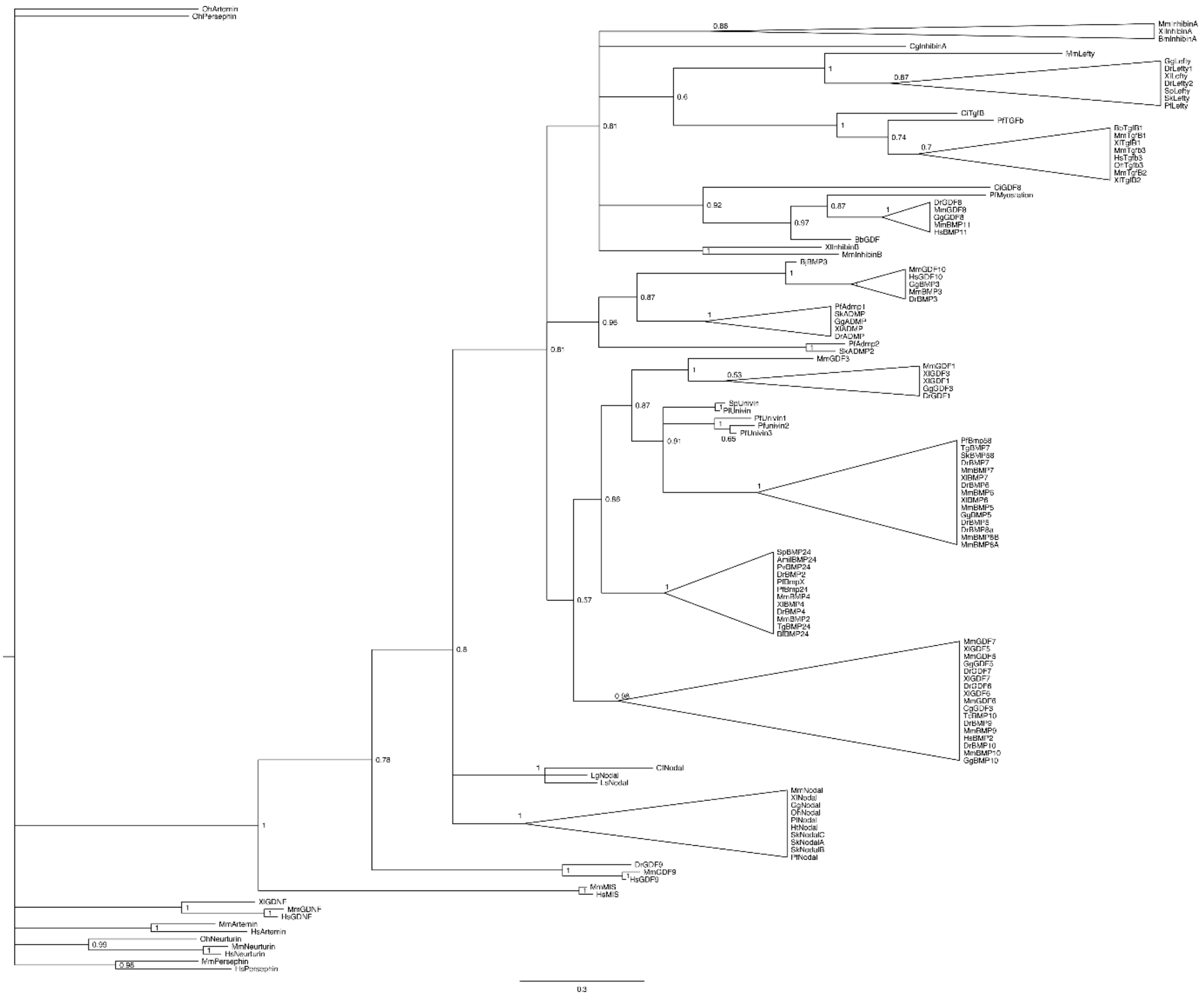

OhArtemin
OhPersephin
0.88
MmInhibinA
XlInhibinA
BmInhibinA
CgInhibinA
MmLefty
1
0.87
GgLefty
DrLefty1
XlLefty
DrLefty2
SpLefty
SkLefty
PfLefty
0.6
CiTgfB
PfTGFb
1
0.74
0.7
BbTgfB1
MmTgfB1
XlTgfB1
MmTgfb3
HsTgfb3
OnTgfb3
MmTgfB2
XlTgfB2
0.81
CiGDF8
PfMyostation
0.92
0.87
1
DrGDF8
MmGDF8
GgGDF8
MmBMP11
HsBMP11
0.97
BbGDF
XlInhibinB
MmInhibinB
BjBMP3
1
MmGDF10
HsGDF10
GgBMP3
MmBMP3
DrBMP3
0.87
PfAdmp1
SkADMP
GgADMP
XlADMP
DrADMP
0.96
PfAdmp2
SkADMP2
MmGDF3
0.53
MmGDF1
XlGDF3
XlGDF1
GgGDF3
DrGDF1
0.87
SpUnivin
PfUnivin
PfUnivin1
Pfunivin2
PfUnivin3
0.65
0.91
PfBmp58
TgBMP7
SkBMP58
DrBMP7
MmBMP7
XlBMP7
DrBMP6
MmBMP6
XlBMP6
MmBMP5
GgBMP5
DrBMP5
DrBMP8a
MmBMP8B
MmBMP8A
0.86
SpBMP24
AmilBMP24
PvBMP24
DrBMP2
PfBmpX
PfBmp24
MmBMP4
XlBMP4
DrBMP4
MmBMP2
TgBMP24
BfBMP24
0.57
MmGDF7
XlGDF5
MmGDF5
GgGDF5
DrGDF7
XlGDF7
DrGDF6
XlGDF6
MmGDF6
CgGDF3
TcBMP10
DrBMP9
MmBMP9
HsBMP2
DrBMP10
MmBMP10
GgBMP10
0.98
0.8
0.78
CiNodal
LgNodal
LsNodal
MmNodal
XlNodal
GgNodal
OfNodal
PlNodal
HtNodal
SkNodalC
SkNodalA
SkNodalB
PfNodal
DrGDF9
MmGDF9
HsGDF9
MmMIS
HsMIS
XlGDNF
MmGDNF
HsGDNF
MmArtemin
HsArtemin
OhNeurturin
0.99
MmNeurturin
HsNeurturin
MmPersephin
0.96
HsPersephin
0.3

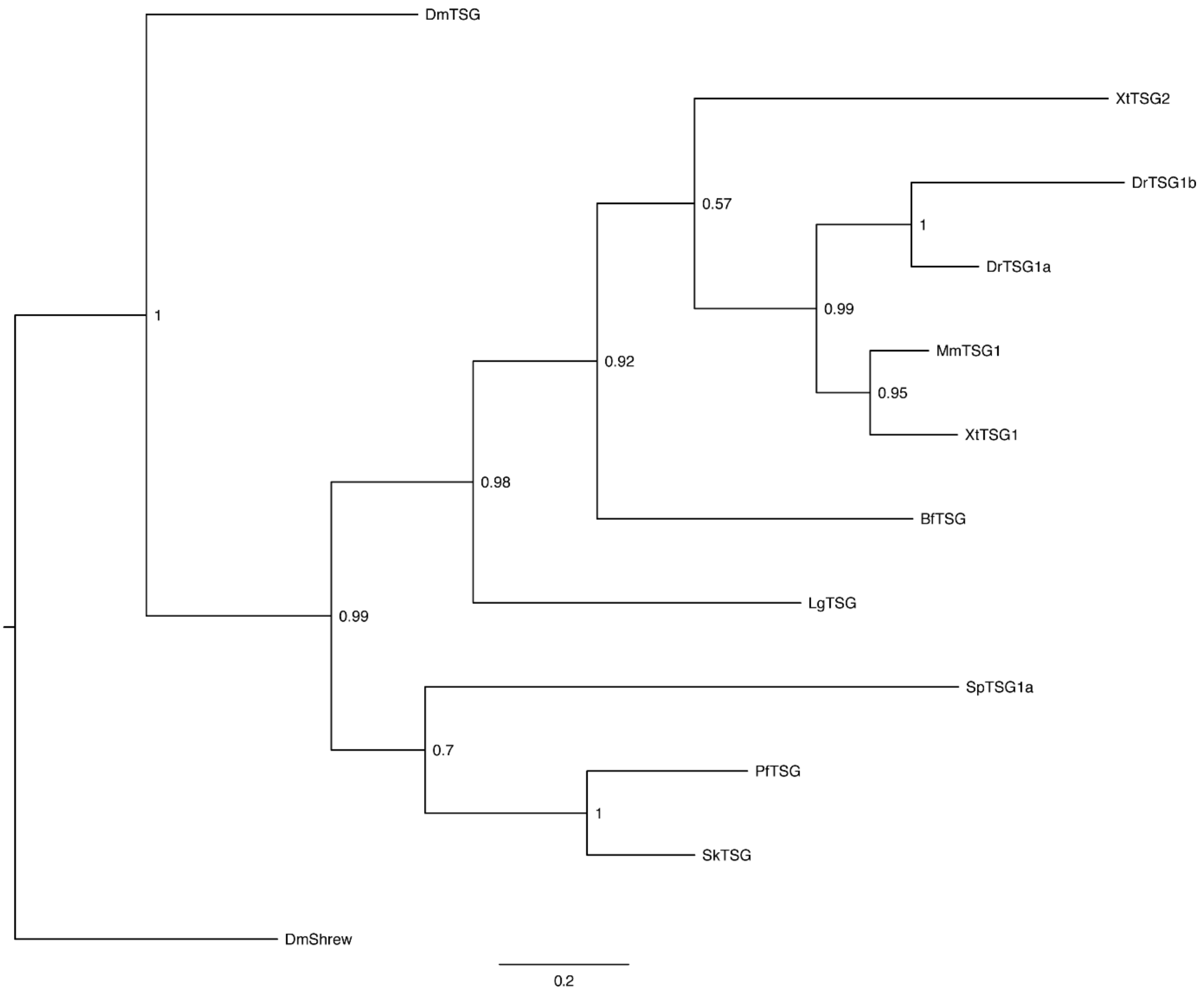
DmTSG
XtTSG2
DrTSG1b
1
DrTSG1a
0.57
0.99
MmTSG1
0.95
XtTSG1
0.92
BfTSG
0.98
LgTSG
1
0.99
SpTSG1a
0.7
PfTSG
1
SkTSG
DmShrew
0.2

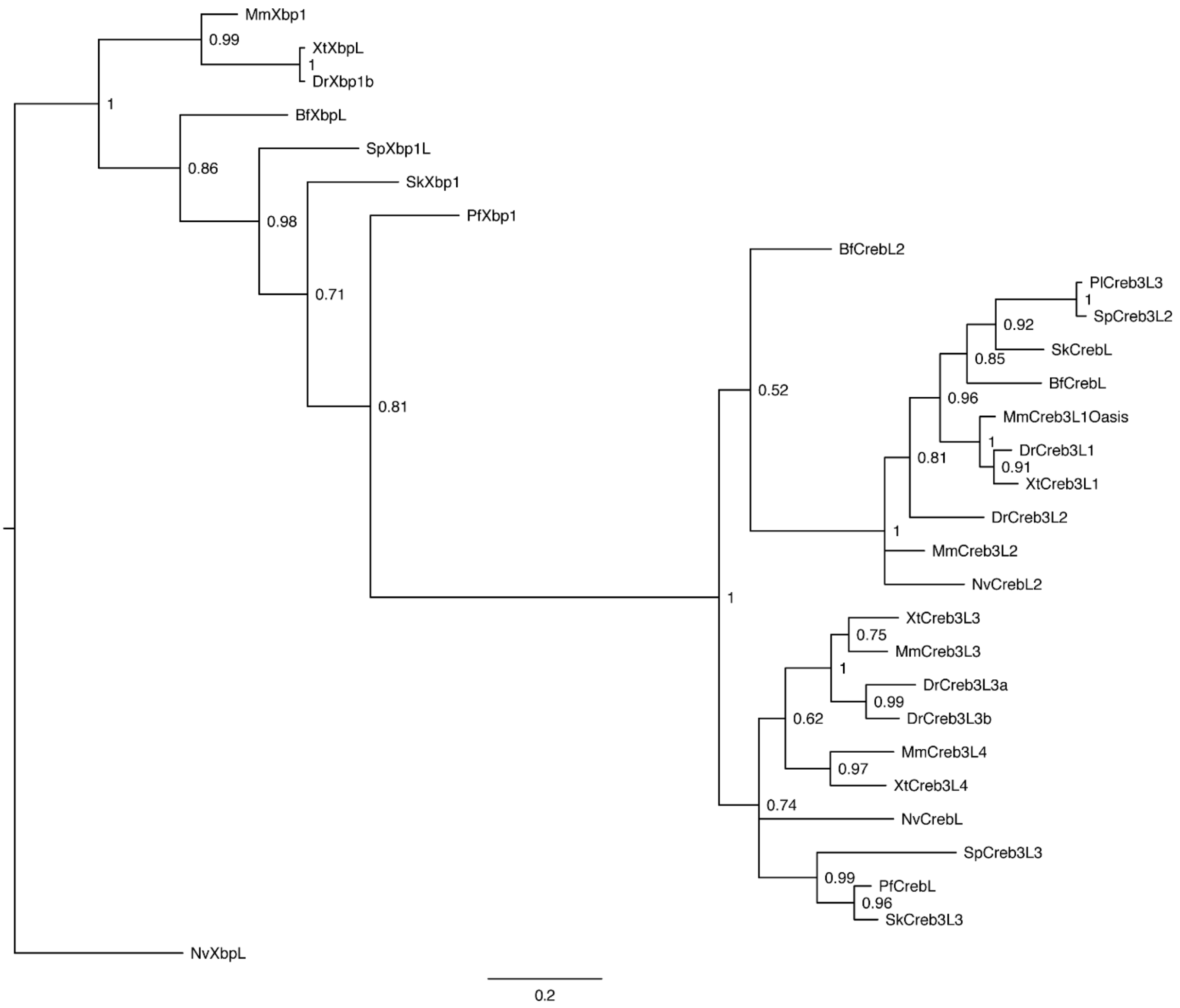
MmXbp1
0.99
XtXbpL
1
DrXbp1b
1
BfXbpL
0.86
SpXbp1L
0.98
SkXbp1
0.71
PfXbp1
0.81
BfCrebL2
PlCreb3L3
1
SpCreb3L2
0.92
SkCrebL
0.85
BfCrebL
0.96
MmCreb3L1Oasis
1
DrCreb3L1
0.91
XtCreb3L1
0.81
DrCreb3L2
1
MmCreb3L2
NvCrebL2
0.52
1
XtCreb3L3
0.75
MmCreb3L3
1
DrCreb3L3a
0.99
DrCreb3L3b
0.62
MmCreb3L4
0.97
XtCreb3L4
0.74
NvCrebL
SpCreb3L3
0.99
PfCrebL
0.96
SkCreb3L3
NvXbpL
0.2

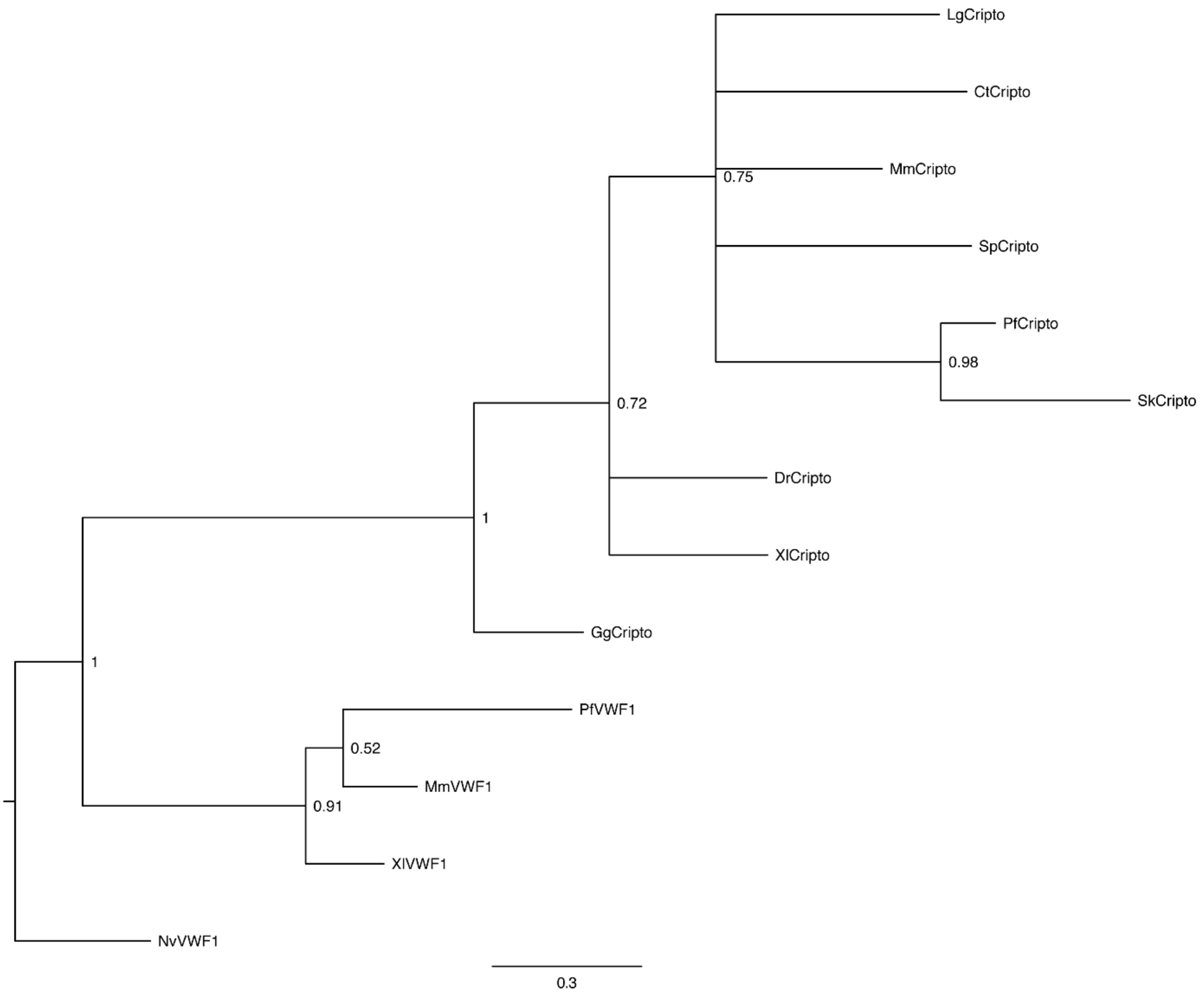

Figure S1. Phylogenetic analysis of newly identified hemichordate genes. Phylogenetic trees were analysed using Bayesian inference of conserved protein domains aligned using MUSCLE. Trees were run through 5 million generations and are based on a “mixed” model of evolution. Domains used in tree construction were identified using SMART protein prediction analysis.

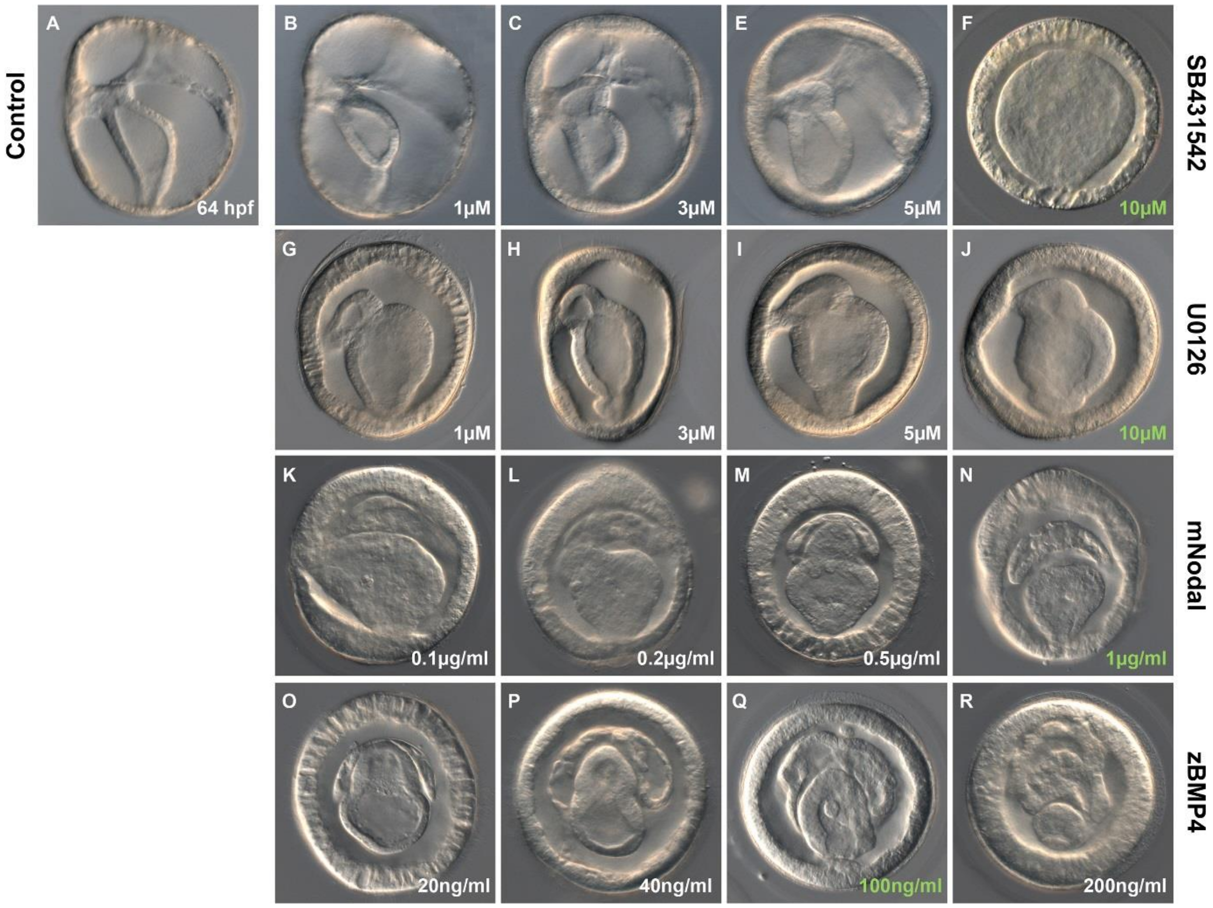

Figure S2. Dose dependent effects of SB431542, U0126, mNodal and zBmp4 treatments. Treatments were initiated at the 2-cell stage at various concentrations to determine the most reliable concentration of a given substance. Green indicates the concentration used for the present study.

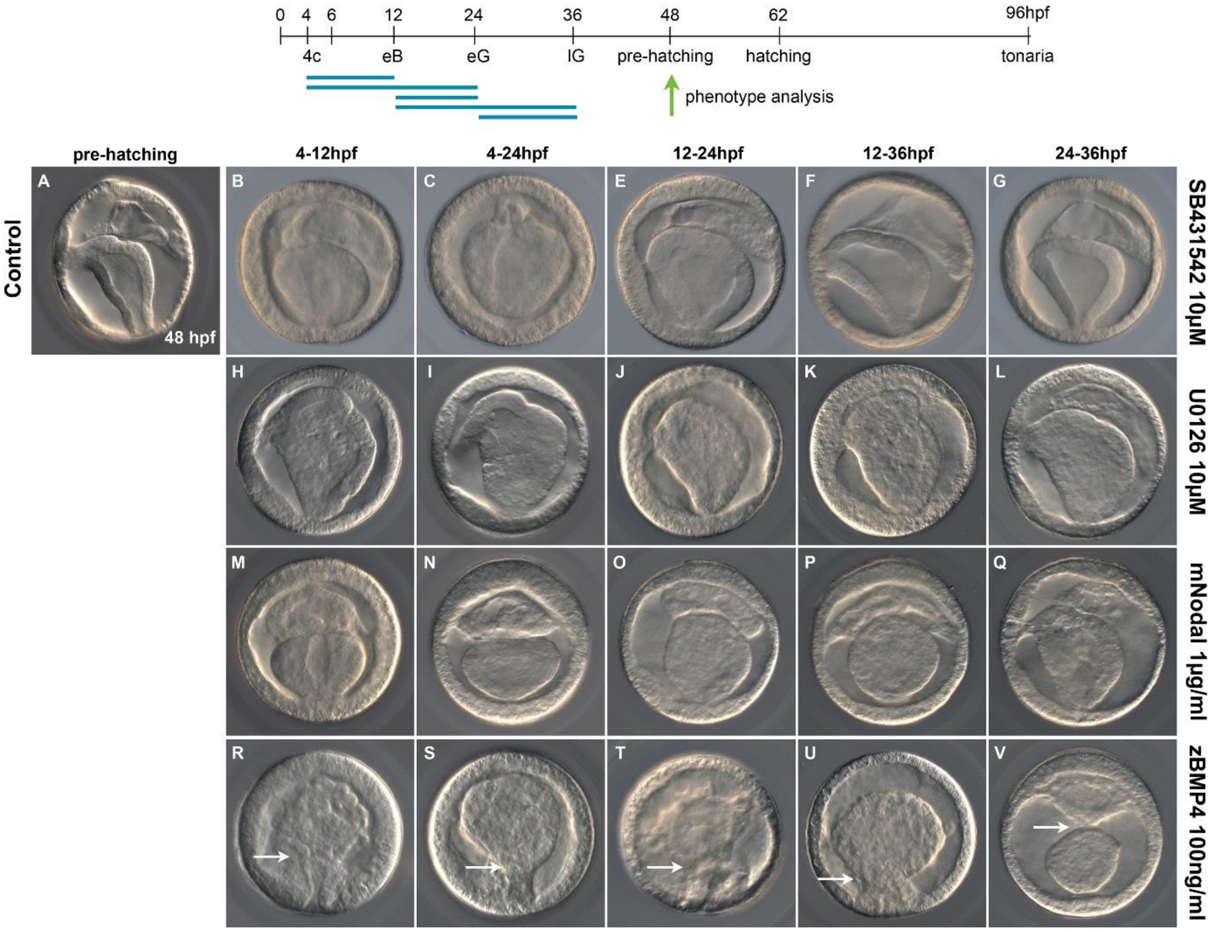

Figure S3. Drug sensitivity-periods of the various treatments in *P. flava*. Embryos were incubated with the indicated drug or recombinant protein during the indicated periods of times and scored for phenotypes at pre-hatching embryos. White arrows in R-V indicate the boundary between the endoderm and mesoderm.

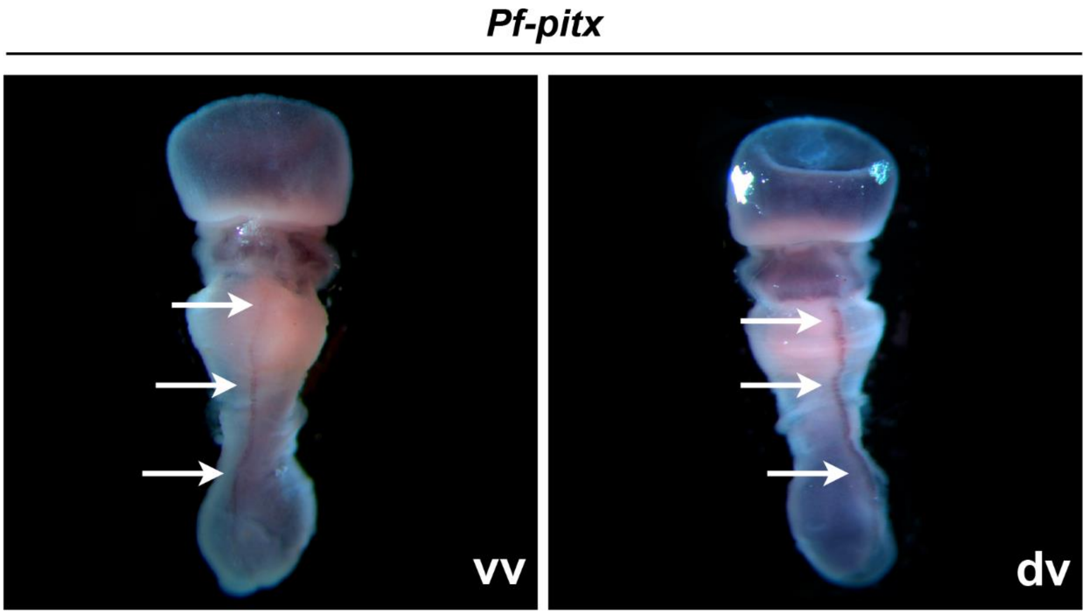

Figure S4. *Pf-pitx* is expressed in the ventral and dorsal midlines. WISH using a probe targeted against endogenous *Pf-pitx* transcripts shows that this gene is expressed in the ventral and dorsal midlines in newly metamorphosed juveniles. (dv) dorsal view, (vv) ventral view.

Table S1. Primers used to clone the full-length *Pf-nodal* gene

degenerated NODAL primers:
Nodal degF1
5'-GTCGACTTCRANMARATHGGNTGG-3'
Nodal degF2
5'-TAYAAYGCNTAYCGNTGYGARGG-3'
Nodal degR1
5'-RCANCCRCAYTCNTCNACDATCAT-3'
Nodal degR2
5'-TTCATRTANGCR TGRTTNGTNGG-3'

NODAL Race primers:
PfNodal_ 3'RACE-Fwd1
5’-GTCCCTGAACCGTGGAGATTTAGTG-3’
PfNodal_3'RACE-Fwd2
5’-AGGTTCAGAGAGACCGCCATCATTC-3’
PfNodal_5'RACE-Rev1
5’-TTTCCCGCCCTTGACCTTGGTGAAG-3’
PfNodal_5'RACE Rev2
5’-GTTGCCGTTTCTTCGTTTCTTTCCCC-3’

Full length NODAL primers:
PfNodal_FL-FWD
5’-ATGGTCCTCTGCCCTCTTTGTGTCG-3’
PfNodal_FL REV
5’-TTATCTGCAACCACATTCTTCCAC-3’
